# Toward Predictive Theory in Single‐Atom Catalysis

**DOI:** 10.1002/advs.75156

**Published:** 2026-04-03

**Authors:** Andrea Ruiz‐Ferrando, Sharon Mitchell, Núria López, Javier Pérez‐Ramírez

**Affiliations:** ^1^ Institute for Chemical and Bioengineering Department of Chemistry and Applied Biosciences ETH Zurich Zürich Switzerland; ^2^ NCCR Catalysis Zürich Switzerland; ^3^ Institute of Chemical Research of Catalonia (ICIQ‐CERCA) The Barcelona Institute of Science and Technology (BIST) Tarragona Spain

**Keywords:** catalyst stability and safety, density functional theory, operando spectroscopy, predictive modeling, single‐atom catalysis, synthesis‐structure‐performance relationships

## Abstract

Single‐atom catalysis has become a central framework for experiment‐theory integration, as catalytic performance is highly sensitive to the environment of individual metal atoms, a feature that electronic structure calculations are well‐suited to analyze. Yet much of current theoretical practice relies on simplified single‐site models and narrow reactivity windows, overlooking the intrinsic site diversity and evolution of single‐atom catalysts (SAC). This Perspective discusses how SAC modeling can be reframed through a lifecycle‐oriented view that integrates synthesis, activity, stability, and safety. By adopting ensemble‐based descriptions and modular thermodynamic descriptors, we show how theory can be used systematically in line with the level of structural definition accessible experimentally. Using acetylene hydrochlorination as a prominent SAC application with exceptional data coherence for examining the theory‐experiment interplay, wedemonstrate that site formation and evolution under synthesis and reaction conditions, as well as ensemble‐driven activity trends consistent with experimental yields, can be treated quantitatively. In contrast, stability and safety are more effectively addressed through comparative, pathway‐resolved analyses. More broadly, this perspective points toward a shift in how SAC modeling is framed across reactions, enabling theory to move beyond post‐rationalization toward disciplined prediction.

## Graphical abstract

A lifecycle‐oriented framework reframes modeling in single‐atom catalysis. By combining ensemble‐based descriptions with explicit validation against experimental observables, the approach defines the scope of theory across catalyst synthesis, activity, stability, and safety, clarifying where quantitative insight is possible and where interpretation must remain qualitative.

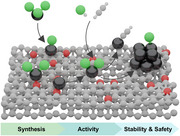

## 1 Introduction

Density functional theory (DFT) has become a key tool in heterogeneous catalysis for connecting atomic‐scale structure with catalytic function [1, 2, 3]. Advances in computational approaches have progressively extended first‐principles modeling from idealized extended surfaces [4] toward increasingly complex catalytic motifs, including supported metal clusters [5, 6] and single‐atom catalysts (SAC) [7, 8], reflecting a growing ambition to describe complex catalytic phenomena [9]. While homogeneous catalysts benefit from well‐defined active sites that enable semi‐quantitative modeling [10], heterogeneous catalysts typically present distributions ofpotentially active species and complex surface environments that constrain theory largely to qualitative descriptions [11]. SAC occupy a distinctive position between these extremes. Isolated metal atoms anchored to supports, and in some cases stabilized by ligands [12], enable atomistic descriptions rarely accessible in heterogeneous catalysis [11], and their rapid development across catalytic regimes [12, 13, 14], has coincided with a surge in computational studies, now accounting for more than half of the SAC literature [15].

DFT has played a central role in rationalizing active‐site structures and catalytic mechanisms in single‐atom catalysis by enabling systematic and controlled exploration of metal coordination environments and adsorbate configurations that are difficult to access experimentally [16, 17]. As the field matures, however, expectations have shifted toward predictive insight that can inform catalyst performance more directly, including quantitative structure‐activity trends and anticipation of catalyst deactivation. However, as revealed by analysis of the SAC literature (vide infra), these expectations are rarely met [18]. This shortfall reflects not only limitations in current modeling practices, but also intrinsic features of SAC, which exist as heterogeneous and evolving ensembles of coordination environments that are typically accessed only indirectly through ensemble‐averaged spectroscopic observables [19, 20, 21, 22]. As a result, establishing a unique and unequivocal mapping between experimental signatures and atomistic models is inherently challenging.

In response, theory has largely relied on idealized single‐site representations assumed to stand in for these ensembles, often with limited experimental constraint and without explicitly accounting for structural evolution during synthesis or operation. Together, these factors confine simulations to predominantly qualitative or retrospective roles [16], obscuring both the reliability of existing predictions and the realistic boundaries of predictivity in single‐atom catalysis.

In this Perspective, we discuss how extracting predictive insight from modeling in single‐atom catalysis requires reframing how catalytic questions are posed across the evolution of a catalytic material. Addressing this challenge calls for a lifecycle‐oriented view that treats site formation during synthesis, its evolution during operation, and the pathways governing stability and safety as interconnected aspects of a single problem. Within this view, ensemble‐based, thermodynamics‐driven descriptions provide a practical level of abstraction, enabling trends to be assessed consistently across structurally diverse SAC while remaining aligned with the experimental resolution accessible under realistic conditions. By making explicit how these theoretical descriptors are constructed and benchmarked against experimental observables [23, 24], we clarify which aspects can be treated quantitatively, where interpretation must remain qualitative, and how far predictive insight can be claimed with confidence at each stage.

We center this Perspective on acetylene hydrochlorination (AH), an industrially relevant reaction traditionally catalyzed by mercury and historically responsible for over 80 % of global mercury emissions [25]. Efforts to replace mercury‐based catalysts in polyvinyl chloride production have led to the exploration of a wide range of alternative catalyst classes. Among these, SAC have emerged as the most effective mercury‐free systems reported to date, with gold‐ and platinum‐based materials on activated carbon exhibiting particularly high performance [26, 27], and ruthenium and copper also investigated [28, 29, 30]. As a result, AH now offers a uniquely mature context for critically examining the framing and practical limits of modeling in single‐atom catalysis [30, 31, 32].

Using this reaction as a guiding example, we combine a systematic analysis of the SAC literature with the lifecycle‐aware modeling perspective introduced above. Under this representation, coordination environments can be captured quantitatively, activity trends can be resolved in terms of relative activity regimes across catalysts, and deactivation pathways and safety considerations can be addressed at a qualitative yet mechanistically consistent level. Beyond AH, we outline the experimental consistency and methodological transparency required to extend similar analyses to other reactions catalyzed by SAC and delineate the conditions under which modeling can meaningfully move beyond retrospective interpretation toward predictive use.

## 2 Assessing Theoretical Practice in SAC

### 2.1 SAC Complexity and Experimental Coherence

Scrutinizing DFT practices requires a system in which experimental complexity is sufficiently resolved to meaningfully evaluate theoretical approaches. Among major SAC applications (Tables S1 and S2), AH offers a uniquely coherent context for examining theory‐experiment integration in single‐atom catalysis. The reaction of acetylene with hydrogen chloride to form vinyl chloride monomer proceeds through a single‐product network, ensuring mechanistic tractability, and has been systematically investigated across multiple metals using broadly comparable synthesis and testing protocols. This experimental foundation is further supported by operando spectroscopic studies that resolve coordination dynamics and deactivation pathways, yielding a level of mechanistic clarity that remains uncommon in single‐atom catalysis (Table S1) [26, 27, 28, 31].

Experimental coherence provides a suitable setting to examine how appropriately theoretical models describe the catalyst and relate to experimentally observed structures. This is particularly important for SAC, where advances in characterization and modeling increasingly allow structures to be described with near‐atomic precision. In practice, however, metal sites often exist as ensembles of closely related coordination environments rather than as a single, uniquely defined configuration. Consequently, interpreting theoretical models and experimental observables requires accounting for this structural diversity when defining structure‐property relationships.

SAC based on carbon supports, for example, inherently span a wide and ill‐defined range of local environments shaped by the nature of carbon defects and surface functionalities [20, 33], and by the coordination chemistry of the metal precursor [34]. Spectroscopic techniques capture this structural complexity, but their signals conflate contributions from multiple sites, making theory essential for relating synthetic conditions to the atomic structures formed and their relative prevalence. These coordination environments can evolve as the catalyst operates, while deactivation arises from multiple intertwined processes affecting these states [34]. In this context, the SAC is better described as an ensemble of plausible local coordination environments formed during synthesis and evolving under reaction conditions. Theory therefore plays a central role in linking synthetic conditions to the atomic structures that are formed, their relative prevalence, and their evolution during catalysis.

Using AH as a representative reaction, we therefore examine whether current DFT practices adequately capture this ensemble‐based and evolving nature of SAC. Our assessment does not address computational accuracy or the correctness of individual mechanistic proposals. Instead, we examine how theoretical models are constructed, what structural assumptions they embed, and how they are used to interpret catalytic behavior and experimental observations.

To structure this analysis, published research articles on AH incorporating DFT simulations (Note S1) are examined across three lifecycle dimensions (synthesis, activity, stability and safety) together with a cross‐cutting validation dimension (Table 1). These criteria capture how atomic models relate to catalyst preparation and structural diversity (synthesis), how catalytic turnover is rationalized, including whether multiple sites and possible structural evolution under reaction conditions are considered (activity), and whether degradation pathways or safety‐relevant intermediates are considered (stability and safety). We finally assess how theoretical predictions are compared with experimental observables (validation) and classify studies according to how these aspects are treated within the modeling framework (Table S3 and Note S1).

**TABLE 1:** Framework for analyzing how DFT models of SAC for acetylene hydrochlorination are constructed and validated. Dimensions follow the catalyst lifecycle, and subdimensions define criteria used to assess modeling assumptions and theory‐experiment consistency.

Dimension	Subdimension	Criteria
Synthesis	Site construction	What types of information are used to construct the catalytic site model?
	Model type	How is the catalyst model represented (cluster or periodic)?
	Site heterogeneity	Are multiple distinct active site structures considered?
	Site formation energies	Is the relative abundance of different sites estimated or discussed?
Activity	Site evolution	Is the structural evolution of the active site during the reaction considered?
	Site contribution	Are the contributions of different sites to activity evaluated?
Stability and safety	Stability analysis	Is catalyst stability or deactivation analyzed using theory?
Validation	Validation reference	Which experimental observables are used to validate the model?

### 2.2 Gaps in Current Theoretical Models of SAC

**FIGURE 1:**
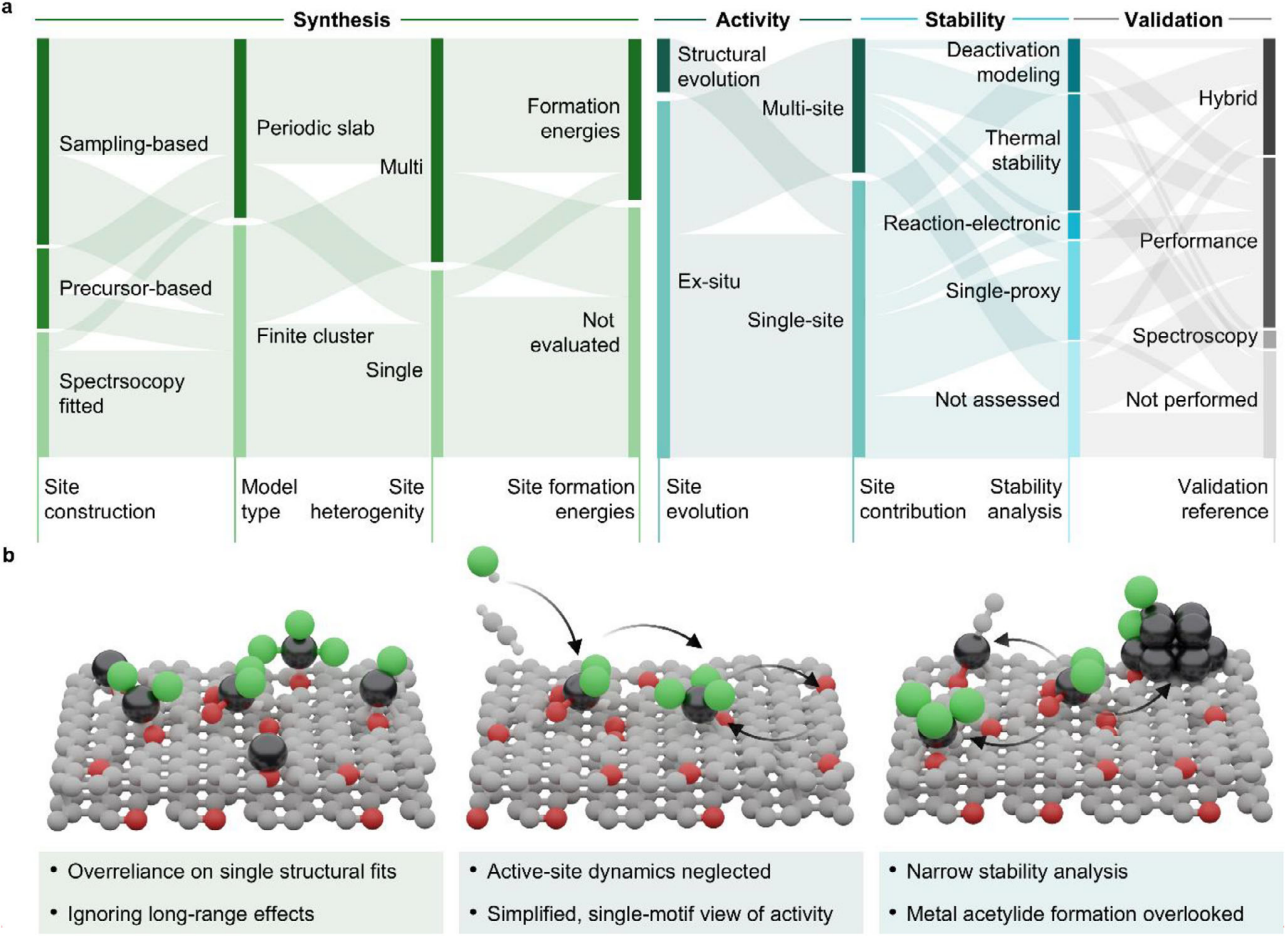
Landscape of DFT modeling practices and gaps in single‐atom catalysis. (a) Alluvial plot showing how published studies on acetylene hydrochlorination address key modeling decisions related to synthesis, activity, and stability, as well as the strategies used to validate theoretical models. Category definitions and evaluation criteria are provided in Note S1 and Table S3. (b) Schematic summary of recurrent limitations in modeling practices, spanning synthesis‐driven heterogeneity, active‐site restructuring, and catalyst deactivation pathways, which are reflective of broader challenges in SAC modeling.

Applying this framework to the AH literature allows systematic comparison of modeling strategies across lifecycle and validation dimensions (Figure 1a; Table S4) and reveals recurring limitations in how relevant questions are addressed (Figure 1b).

At the synthesis stage, the central question concerns which environments emerge from catalyst preparation. In practice, a quantitative link between synthesis conditions, site populations, and theoretical models is rarely established (Table S4). Spectroscopic observations are often used to guide model construction and, in about 20 % of the studies, directly determine the coordination motif considered. Under these practices, the underlying distribution of sites is assumed rather than resolved [35]. This implicitly treats average or effective structures as representative of the material, without evidence that this assumption is warranted.

In other cases, models either retain invariant structures derived from precursor species (30 %) or explore several plausible motifs through structural sampling (50 %). In all cases, however, the formation and evolution of sites during synthesis are not explicitly considered. Although formation energies are reported in about 37 % of the studies, site populations are rarely evaluated, and spectroscopic observations are generally incorporated into model construction rather than used for independent validation. Consequently, the distribution of sites present in the material is typically assumed rather than examined. In addition, 57 % of the studies rely on finite cluster models, truncating the extended carbon environment. Such representations may neglect longer‐range support effects, potentially altering the electronic structure and coordination flexibility of the metal center compared with models that explicitly represent the extended carbon framework.

At the activity stage, the central question is which sites and chemical steps control catalytic turnover under working conditions. Although coordination environments coexist, evolve during reaction [36], and contribute unequally to reactivity [37], site evolution is explicitly considered in only 13 % of studies, and 67 % rationalize activity using a single representative structure. Even when multiple sites are considered, they are evaluated individually rather than treated collectively as an ensemble. In addition, the carbon support is also frequently treated as an inert component of the catalyst, despite evidence that it can act as an acetylene reservoir or even a co‐catalyst [31]. As a result, activity is commonly conceived as a property of a fixed catalytic entity, allowing models to reproduce overall trends while offering limited insight into which sites govern performance or which operando‐observed changes are mechanistically relevant.

The treatment of stability and safety is still more limited, even though deactivation pathways may involve hazardous intermediates. Only 13 % explicitly examine deactivation pathways, few consider all relevant mechanisms, and acetylide formation is rarely discussed. In most cases, thermal stability is considered only indirectly, most often through formation energies (28 %), which are rarely analyzed explicitly, or through descriptors based on adsorbate interactions or electronic properties (24 %). Overall, theory offers little guidance on failure modes, and safety remains disconnected from catalyst design, preventing these considerations from being integrated into rational catalyst development.

Finally, validation practices further weaken the connection between theory and experiment. Comparisons are predominantly qualitative and typically limited to post hoc consistency checks, such as reproducing activity trends (41 %), and are only combined with spectroscopic validation in 28 % of the cases. As a result, agreement with experimental trends is often interpreted as confirmation of model adequacy, but there is no systematic validation of theoretical observations with experimental observables. Moreover, the criteria used to establish such validation, as well as the broader logic guiding model construction, are often far less explicit than the computational parameters themselves.

## 3 Toward a Lifecycle‐Aware Modeling in SAC

Uncertainty is one of the major issues in modeling SAC. Discussions of DFT accuracy typically focus on methodological choices such as the exchange–correlation functional and computational setup. However, the resulting uncertainties are relatively well characterized, typically ∼0.1–0.2 eV for adsorption energies [38], and can be managed through benchmarking. Yet a potentially larger source of uncertainty often receives far less attention.

In SAC systems, uncertainties arising from the structural representation of the catalyst can be much larger. When oversimplified structural models are used (e.g., single‐site descriptions or neglect of site evolution and support effects), plausible coordination environments may be excluded from the model space. Because these environments can exhibit distinct adsorption energetics, calculated energies depend strongly on the structures considered, introducing an uncertainty associated with the spread of energetics across accessible coordination environments whose magnitude is system‐dependent and difficult to bound a priori.

Addressing this source of uncertainty, therefore, requires moving beyond single‐site approximations toward models that explicitly consider ensembles of coordination environments and their evolution across the catalyst lifecycle. On this basis, we adopt a modular modeling strategy built around physically grounded descriptors, in which site formation during synthesis, ensemble behavior under reaction conditions, and pathways of degradation and safety are treated as connected questions and explicitly related to experimentally accessible observables.

Because experimental characterization typically averages stable or metastable configurations rather than uniquely resolved atomic structures, this ensemble description is most naturally formulated within a thermodynamic framework. Such an approach enables tractable exploration of site populations and reactivity trends while remaining consistent with the structural information accessible through spectroscopic characterization.

Within this framework, comparison between theoretical descriptors and corresponding experimental observables allows the accessible ensembles to be progressively refined and the associated uncertainties to be quantified where possible or otherwise delimited, providing a transparent assessment of the attainable scope and reliability of theoretical predictions.

### 3.1 Lifecycle Modeling of Acetylene Hydrochlorination

**FIGURE 2:**
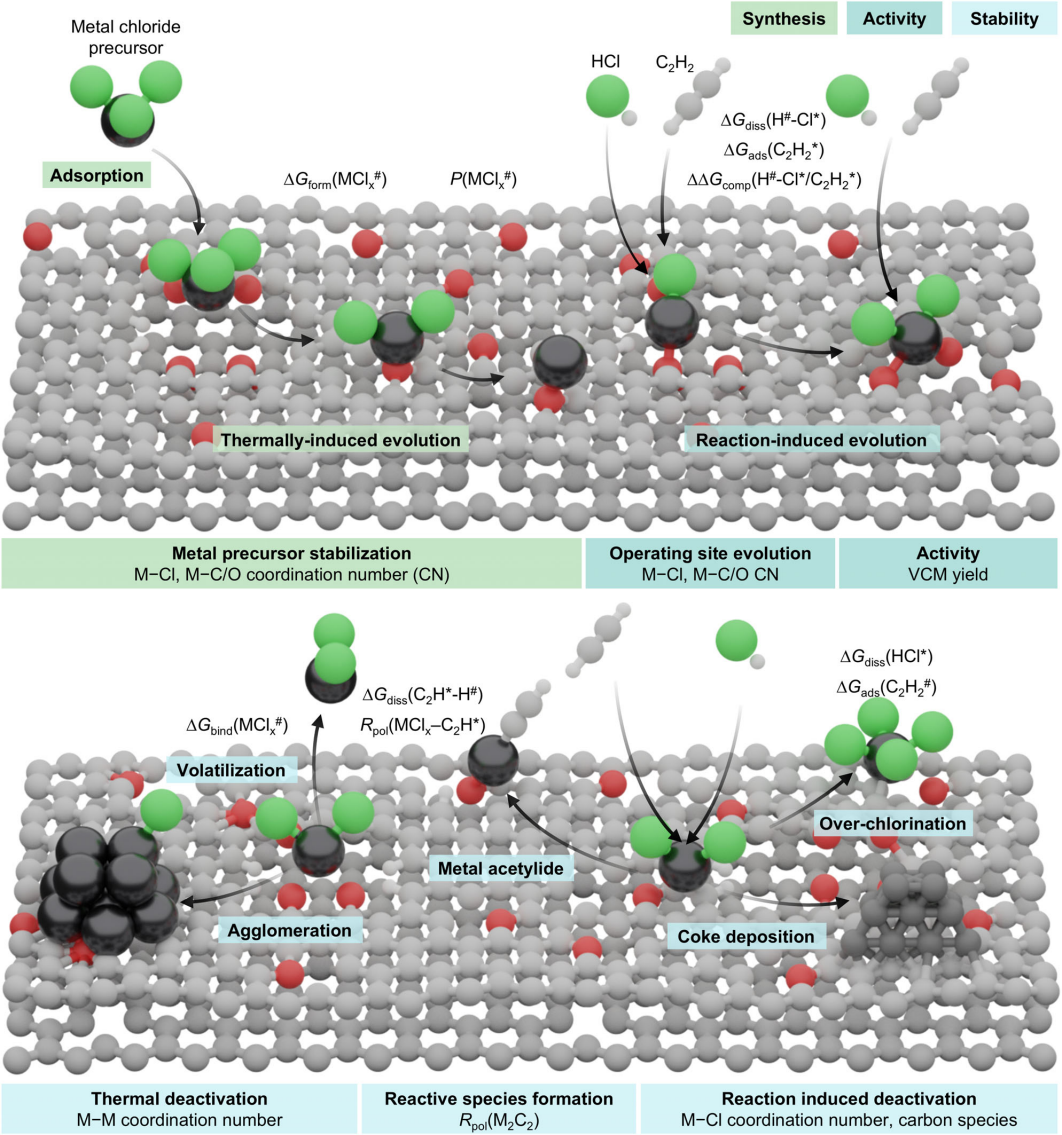
Lifecycle‐aware framework for modeling SAC. The framework organizes catalyst synthesis, activity, stability, deactivation, and safety into a unified thermodynamic and electronic description. Using AH as a benchmark, it illustrates how ensemble‐level descriptors enable systematic comparison across sites and metals, while clarifying where theoretical treatment can be quantitative and where it remains qualitative. Each stage is associated with experimentally accessible observables used for validation. Color legend: carbon (gray), chlorine (green), oxygen (red), hydrogen (white), metal atoms (metallic gray). Descriptor definitions are provided in Table S5.

Within this lifecycle‐oriented perspective, modeling AH begins at the synthesis stage by explicitly separating the two key steps common to SAC preparation: impregnation and thermal activation. Incipient wetness impregnation is represented by chlorinated metal precursors adsorbed on activated carbon, taken as a common and experimentally accessible starting point across metals (Figure 2). Carbon sites are described using selected morphologies and functional groups consistent with available characterization and typical dry and wet impregnation routes (Note S2), capturing recurring metal–support motifs in SAC [13]. Thermal activation is then modeled as progressive ligand depletion, generating families of metal chloride species characterized by their formation free energies, Δ*G*_form_(MCl_x_^#^) (Table S4). Relative populations are obtained from Boltzmann statistics, yielding ensembles that can be directly confronted with X‐ray absorption spectroscopy (XAS) spectra of fresh catalysts.

Catalytic operation is addressed by propagating these ensembles into reactivity analysis. Although activity in SAC is often rationalized solely through metal–acetylene adsorption energies [30, 39], operando XAS analyses indicate that carbon functionalities contribute significantly to acetylene uptake [31], motivating an ensemble‐based description in which activity emerges from competing interactions. Three thermodynamic descriptors capture the key processes governing operation: the dissociation free energy of hydrogen chloride, Δ*G*_diss_(H^#^‐Cl*), the adsorption free energy of acetylene, Δ*G*_ads_(C_2_H_2_*), and their competition, ΔΔ*G*_comp_(H^#−^Cl*/C_2_H_2_*). These descriptors quantify the balance between chlorine transfer and acetylene binding while tracking coordination changes at the metal center, enabling direct comparison with operando XAS and evaluation against vinyl chloride yields across metals.

The analysis then proceeds to catalyst deactivation and safety. Thermal stability is assessed through metal–support binding free energies, Δ*G*_bind_(MCl_x_^#^), and evaluated against XAS signatures of metal–metal bond formation. Acetylene dissociation, Δ*G*_diss_(C_2_H*‐H^#^), is modeled explicitly due to its role in forming unstable metal acetylides, with electronic character quantified by the charge polarization ratio (Note S3), *R*_pol_(MCl_x_─C_2_H*). Over‐chlorination is evaluated via hydrogen chloride dissociation energetics, while coke formation is assessed through acetylene adsorption on the carbon support, Δ*G*_ads_(C_2_H_2_^#^), cross‐validated against Brunauer‐Emmett‐Teller specific surface area measurements and electron paramagnetic resonance spectroscopy.

By assessing it across gold, platinum, ruthenium, and copper SAC in AH, this framework is used to examine how rigorous theory can be applied across synthesis, operation, and deactivation under realistic structural constraints, and to delineate the boundary between quantitative and qualitative modeling in relation to experimental coherence.

### 3.2 Structural Models Guided by Synthesis Constraints

**FIGURE 3:**
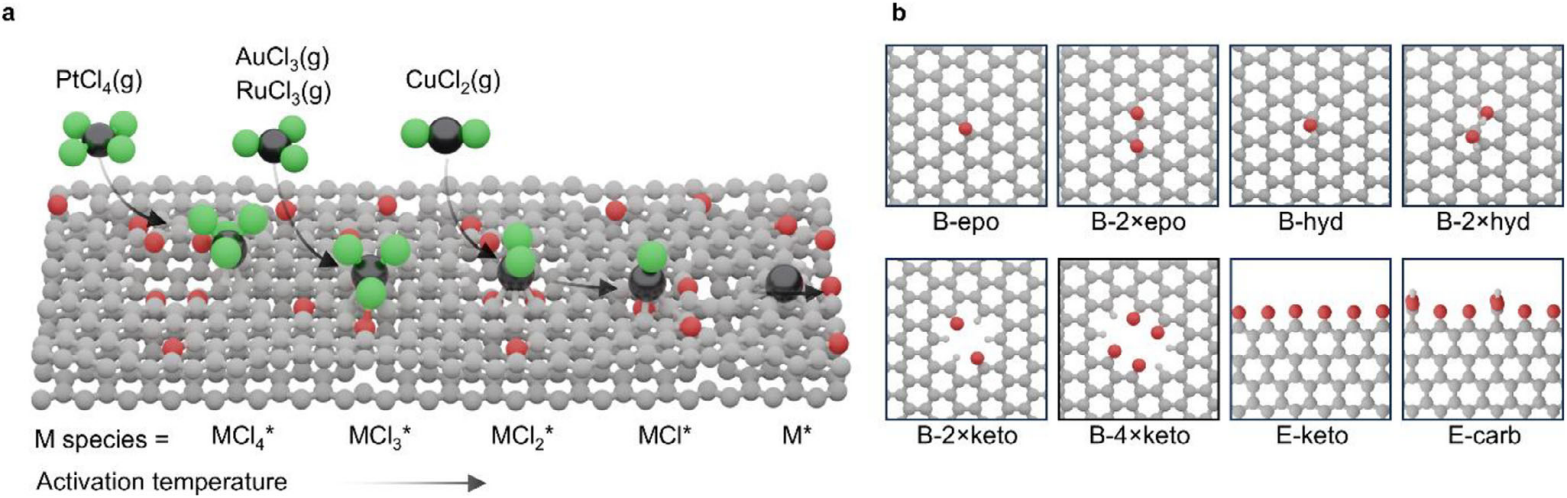
Synthesis‐stage representation of coordination environments in SAC based on carbon supports. (a) Evolution from metal precursor adsorption to ligand depletion and thermal restructuring, leading to ensembles of thermodynamically accessible coordination environments rather than single, defined sites. (b) Carbon motifs used to represent the structural and chemical diversity of activated carbon supports.

We begin by modeling the thermodynamics of metal precursor anchoring on the carbon support during catalyst preparation (Figure 3a) to establish the expected structural ensemble used in subsequent analyses. Metal chloride precursors (AuCl_3_, CuCl_2_, PtCl_4_, and RuCl_3_) were modeled as adsorbed on representative surface motifs that capture the compositional and coordination diversity of activated carbons identified experimentally (Figure 3b, Note S1).

Formation energies of these configurations (Table S6) were used to estimate Boltzmann populations of anchored species (Figure S1). Several motifs yield negligible populations across metals (B‐epo, B‐hyd, B‐2×hyd, and E‐carb), indicating that these environments are thermodynamically disfavored during impregnation and unlikely to host metal species at the low metal contents typical of these catalysts (∼1 wt. %). These motifs were consequently excluded from subsequent calculations.

**FIGURE 4:**
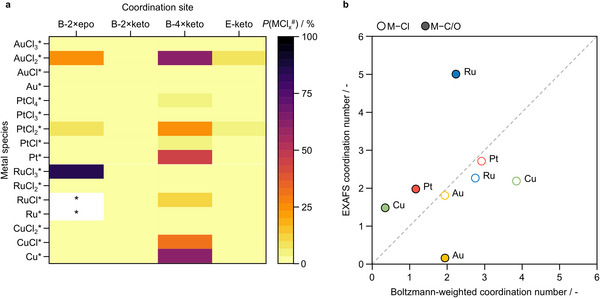
Ensemble description of coordination environments formed during SAC synthesis. (a) Boltzmann populations (*P*(MCl_x_^#^), as %) of (chlorinated) metal species at 473 K, derived from their formation energies, illustrating how synthesis generates distributions of coordination environments; * indicates formation of volatile species. (b) Comparison between Boltzmann‐averaged coordination numbers and EXAFS‐derived values [29, 31], illustrating how ensemble‐level descriptors can be confronted with spectroscopic observables. Color legend: carbon (gray), chlorine (green), oxygen (red), hydrogen (white), metal atoms (metallic gray).

Thermal activation is then considered through stepwise chloride removal, generating families of metal–chloride species (MCl_x_*). Their formation energies and derived populations (Figure 4a; Table S7) describe the distribution of accessible coordination environments. The resulting populations differ markedly across metals, reflecting the combined influence of metal identity, chlorination degree, and carbon support structure.

This procedure yields a synthesis‐derived structural ensemble in which the catalyst is represented not by a single model but by a set of thermodynamically accessible coordination environments, each with an equilibrium population. In practice, the ensemble defines the structural models to be used in subsequent analyses and their expected relative contributions. To assess whether this representation is meaningful, the ensemble is validated against structural information derived by analysis of the extended X‐ray absorption fine structure (EXAFS) by comparing Boltzmann‐averaged metal–chloride and metal–support coordination numbers of the MCl_x_ species with the corresponding experimental values (Figure 4b).

The models reproduce the degree of chlorination comparatively well, with an average deviation of 0.64 coordination bonds for M–Cl coordination, typically within the intrinsic uncertainty of EXAFS coordination number fitting (up to ∼1 bond). Larger discrepancies appear for metal‐support coordination (1.64 bonds), reflecting the greater structural uncertainty associated with the heterogeneous carbon support.

Importantly, deviations from experimental observations are informative signals. The observed Au–Au coordination reflects the tendency of gold to sinter unless stabilized by additional ligands (Table S10), a process not included in this part of the thermodynamic description, which only considers single‐atom configurations. For ruthenium, the Ru–C/O coordination number suggests the presence of more extended coordination environments than those captured by the limited set of carbon motifs considered here.

Across metals, this comparison quantifies the structural error introduced during model construction, an uncertainty that typically remains implicit in conventional modeling approaches based on single‐site representations or on adjusting models to reproduce spectroscopic signatures. Importantly, this allows model‐experiment agreement to emerge naturally, while deviations indicate where the structural model requires improvement. The comparison with EXAFS, therefore, also provides an objective reference to assess whether future modifications of the structural model improve or deteriorate its agreement with experiment.

Within this framework, the structural description at the SAC synthesis stage can therefore be quantitatively assessed at the ensemble level, as its deviation from experiment is explicitly measured. Coordination environments outside this structural space define the limits of the present model. Although not yet standard practice in SAC modeling, thermodynamic ensemble approaches offer a pragmatic and computationally efficient route to contextualize how synthesis shapes active‐site populations, while also making explicit the assumptions and limitations that govern their predictive scope.

### 3.3 Connecting Activity to Ensembles and Their Evolution

Building on the ensemble‐based structural view, catalytic activity is then examined in terms of how coordination populations emerging from synthesis evolve and operate under reaction conditions. The synthesis‐derived models therefore serve as the starting point for the catalytic analysis, where each environment is evaluated individually and updated as it evolves under reaction conditions. The objective is to identify activity regimes rather than predict absolute catalytic rates, as the analysis remains within a thermodynamic descriptor framework.

**FIGURE 5:**
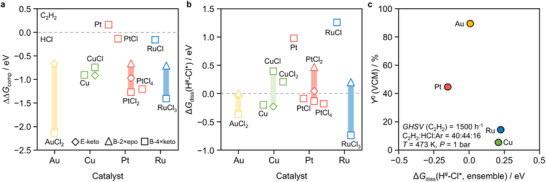
Connecting structure and activity through ensemble‐level adsorption descriptors. (a) Gibbs free energy differences for competitive adsorption of hydrogen chloride vs. acetylene on the most populated species, illustrating the partitioning of reactivity between metal and carbon environments. (b) Gibbs free energies for hydrogen chloride dissociation across the most populated species, representing how synthesis‐derived ensembles evolve under reaction conditions. (c) Relationship between the Gibbs free energy of hydrogen chloride dissociation evaluated for the ensemble of evolved sites and vinyl chloride monomer yield, illustrating how catalytic performance reflects collective properties of the operating ensemble rather than isolated sites.

Because hydrogen chloride and acetylene compete for interaction with the metal center, the analysis begins by evaluating their competitive adsorption to identify metal‐dependent preferences at the active site. Competitive adsorption reveals that, across the most populated states (population >2 % in the synthesis‐derived ensemble, Figure 5a) and the full set of configurations (Table S8), metal sites preferentially activate hydrogen chloride rather than bind acetylene, in agreement with related studies [31]. Hydrogen chloride dissociation, therefore, emerges as the key descriptor governing both the evolution of coordination environments and catalytic turnover differences between metals.

Site evolution toward the ensemble of metal coordination environments present under reaction conditions is examined through the exergonicity of hydrogen chloride dissociation, which reflects the thermodynamic driving force for chloride accumulation at the metal center (Figure 5b). Although the distribution is strongly site‐dependent, clear trends emerge across metals. Ruthenium shows the most exergonic dissociation energies, platinum occupies an intermediate regime with a larger fraction of exergonic configurations, whereas gold and copper display weaker and more balanced distributions, indicating no clear tendency toward chlorination.

To analyze catalytic turnover, the ensemble is updated to reflect this evolution. Coordination environments are evaluated individually within the ensemble rather than through ensemble averages, as catalytic turnover does not necessarily scale with thermodynamic population, although low‐population environments are excluded here for tractability (below 2 %, Note S2). The remaining configurations are updated when hydrogen chloride dissociation is sufficiently favorable (Δ*G*_diss_(H^#^‐Cl*) < −0.4 eV), generating an ensemble that incorporates thermodynamically accessible chlorination states (Note S2).

Activity is then evaluated through hydrogen chloride dissociation energetics across this updated ensemble. Configurations with strongly endergonic dissociation (Δ*G*_diss_(H^#^‐sCl*) >0.4 eV, Note S2) are excluded as turnover‐relevant environments (Figure 5c). Catalytic behavior is interpreted in terms of the proximity of dissociation energies to thermoneutrality. Gold displays a narrow distribution centered close to thermoneutral values, whereas platinum spans a broader but still near‐thermoneutral range. In contrast, ruthenium and copper occupy more constrained regions further from thermoneutral conditions, indicating more favorable activity regimes for gold, intermediate regimes for platinum, and less favorable regimes for ruthenium and copper.

These trends are validated in two stages to determine the scope of theoretical insight into catalytic activity. First, the predicted chlorination tendencies agree with operando EXAFS observations (Table S9), which show increased M─Cl coordination for ruthenium and platinum and smaller or negative changes for gold and copper. Site evolution is therefore interpreted qualitatively. Hydrogen chloride dissociation energies capture the thermodynamic driving force toward chlorination but do not define clear limits between strongly and weakly chlorinated environments. The descriptor is thus used to identify tendencies and update the synthesis‐derived ensemble for activity analysis.

Second, the predicted ordering of activity trends is consistent with the experimentally observed ordering of vinyl chloride yield across metals (Figure 5c), although differences between ruthenium and copper remain difficult to resolve. Because energetic thresholds define turnover‐relevant coordination environments, catalytic behavior can be organized into activity regimes at a semi‐quantitative level. While absolute catalytic rates are not predicted, the descriptor provides a quantitative basis to delimit catalytically relevant regimes and interpret activity trends across metals, within the structural resolution established in the synthesis analysis.

Within this framework, theory is not used to identify a unique active site or to impose a predefined reaction pathway. Instead, it evaluates how coordination environments within the evolving ensemble are distributed under working conditions and how this distribution biases catalytic performance. Importantly, the relevant energetic window is relatively narrow, indicating that several coordination environments remain thermodynamically accessible under reaction conditions, an effect that cannot be captured within conventional single‐site representations.

This assessment provides a robust way to interpret activity trends without embedding mechanistic assumptions from the outset. In practice, this allows theory to inform which coordination motifs and metal–support interactions are most relevant for performance, and therefore where synthesis efforts are most likely to be impactful.

### 3.4 Anticipating Instability and Safety Risks

When stability and safety are considered through a lifecycle‐oriented lens, deactivation in AH can be decomposed into dominant degradation modes rather than treated as a single loss‐of‐activity outcome (Figure 5). The principal processes reported include loss of dispersion, chlorine accumulation, and carbon deposition, while safety concerns arise from the potential formation of explosive metal acetylides under acetylene‐rich conditions [28, 31, 40, 41]. These processes are therefore analyzed individually using targeted theoretical descriptors linked to experimentally accessible observables.

**FIGURE 6:**
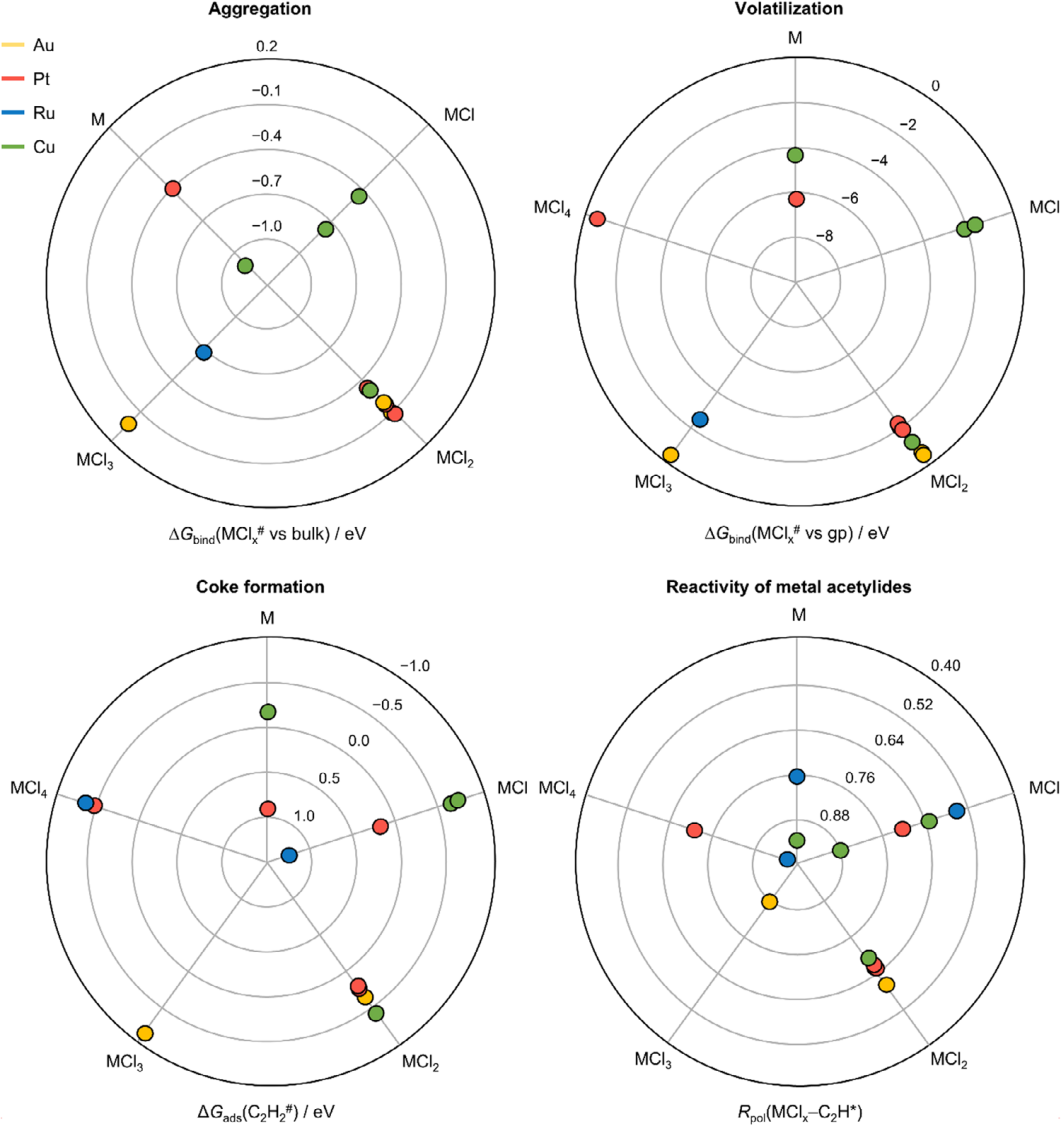
Framework for assessing metal‐dependent stability and safety risks in SAC. Stability and safety are evaluated using five complementary criteria that capture distinct deactivation and risk pathways. Metal–support binding free energies indicate susceptibility to volatilization or aggregation. Coke formation is assessed through acetylene adsorption on carbon. The ionicity of metal acetylide intermediates, derived from charge density differences, captures metal–acetylide interaction strength and potential hazard associated with acetylide formation.

Thermal stability associated with loss of dispersion is assessed using thermodynamic descriptors based on the binding energies of metal chloride species relative to gas‐phase and bulk references, which capture volatilization and aggregation tendencies. Within this description, gold species display significantly weaker stabilization on the support than platinum, ruthenium, and copper (Figure 6), facilitating detachment from carbon defects and subsequent formation of gold clusters. This trend is consistent with operando EXAFS measurements showing substantial Au─Au coordination under reaction conditions, whereas the other metals largely preserve atomic dispersion (Table S10).

Over‐chlorination is not captured as an independent degradation pathway in the present model. Hydrogen chloride dissociation energetics and the thermodynamic driving force for site chlorination already determine the chlorination states present in the ensemble under reaction conditions and do not allow distinguishing between normal site evolution and over‐chlorination. From an experimental perspective, the same limitation applies: the observable chlorination states reflect the equilibrium distribution of sites under reaction conditions. Consequently, chlorination is represented within the structural evolution of the ensemble but cannot be isolated as a separate deactivation mechanism.

Coke formation is assessed through acetylene adsorption on carbon species adjacent to the metal sites, reflecting the preferential interaction of the metal center with hydrogen chloride and consistent with electron paramagnetic resonance observations suggesting that coke formation occurs primarily on the carbon support [31]. Adsorption energies span a similar range across metals, indicating that the initial adsorption step alone does not differentiate between catalysts (Figure 6). Experimentally, however, catalyst deterioration differs markedly (Table S11): ruthenium exhibits the largest surface‐area loss, consistent with more extensive carbon deposition, whereas copper remains essentially unchanged. This lack of correlation suggests that coke formation involves processes within the carbon support that are not captured by the adsorption events considered in the present model, or reflects uncertainties in the structural representation of the carbon matrix.

Safety considerations are evaluated through the thermodynamic accessibility of acetylide formation (acetylene dissociation on the metal) and the electronic character of the resulting metal–acetylide bond. In general, acetylide formation is not thermodynamically favored within the explored coordination environments. However, if formed, copper acetylide species display stronger polarization of the metal–acetylide bond (Figure 6), indicating a more ionic acetylide character. Because direct experimental observables for these intermediates are currently lacking, the results are compared with reference non‐SAC acetylides (Table S12), where copper also exhibits the highest ionic character. At the same time, the emergence of new coordination environments in SAC suggests that previously unobserved reactivity patterns cannot be excluded and should be further investigated from a safety perspective [40, 41, 42, 43, 44, 45].

Taken together, stability and safety are reframed not as generic loss of activity, but as relative propensities of operating ensembles toward specific degradation or hazard pathways. Rather than asking whether a catalyst deactivates, theory is used to identify which failure modes are statistically favored for a given metal–support system and which experimental observables are most diagnostic under working conditions. Thermodynamic descriptors capture trends in metal–support stabilization and qualitatively rationalize the experimentally observed loss of dispersion, which emerges as the most clearly identifiable stability limitation. By contrast, chlorine accumulation, coke formation, and acetylide formation remain less resolved, partly because the intertwined nature of these processes makes it difficult to isolate them individually, even when specific spectroscopic signatures are available, highlighting the need for further combined experimental and theoretical investigation of catalyst deactivation.

## 4 Toward Generalizable Frameworks for Modeling SAC

Within the lifecycle‐aware framework proposed here, synthesis outcomes can be treated quantitatively because thermodynamically derived structural ensembles have bound numerical uncertainties in their relative energies and populations. Catalytic activity is interpreted semi‐quantitatively from the relative energetics of catalytically competent coordination environments within the ensemble under reaction conditions, allowing activity regimes to be distinguished without requiring absolute rate predictions. Stability and safety, in contrast, remain qualitative because the intertwined nature of degradation pathways and the remaining structural and mechanistic uncertainties limit the analysis to identifying comparative tendencies and performance‐limiting regimes.

Although illustrated here using AH as a benchmark, the phenomena discussed are not confined to a single reaction. Structural heterogeneity, the coexistence and evolution of multiple site families, intertwined deactivation pathways, and safety‐relevant transformations recur broadly across single‐atom catalysis, spanning different metals, supports, and synthesis routes. Likewise, many of the controlling factors considered here, such as site formation tendencies, thermal stability, aggregation, and metal–ligand affinities (e.g., chlorophilicity or oxophilicity), reflect general features of metal–support chemistry rather than reaction‐specific peculiarities.

The framework is therefore not restricted to SAC based on carbon supports and can be extended to systems involving other supports and reactions. Its transferability lies not in the specific descriptors introduced for AH, which must be adapted to the chemistry of each system, but in the modeling logic that links synthesis‐derived site ensembles, catalytic operation, and deactivation within a unified description validated against experimental observables.

The extent to which structural uncertainty affects the analysis depends strongly on the nature of the support and the relevance of coordination ensembles. Carbon supports exhibit structural variability arising from defects, functional groups, and edge sites, making ensembles of possible active sites particularly important. In contrast, oxide and nitride supports are governed by crystallographic terminations, lattice defects, and local electronic structure effects that shape the coordination and reactivity of the metal center [46].

Validating modeling strategies requires coherent datasets that enable interrogation across all stages, including harmonized synthesis and testing protocols across metals, operando techniques capable of tracking site evolution under working conditions, post‐reaction analyses that disentangle distinct deactivation pathways, and identification of reactive or hazardous intermediates formed under realistic conditions.

Within this context, safety must be treated as an explicit and integral dimension of catalyst assessment. This requires the establishment of shared interpretations for stability‐ and safety‐relevant descriptors, which must be evaluated relative to boundaries separating normal operation from regimes associated with degradation or hazard. In AH, acetylide formation defines such a boundary; analogous limits are expected in other SAC applications, including hydride or nitride formation in ammonia synthesis, uncontrolled halogen cycling in oxychlorination, or radical accumulation in oxidative and electrocatalytic environments (Table S1) [40, 41, 42, 43, 44, 45].

**FIGURE 7:**
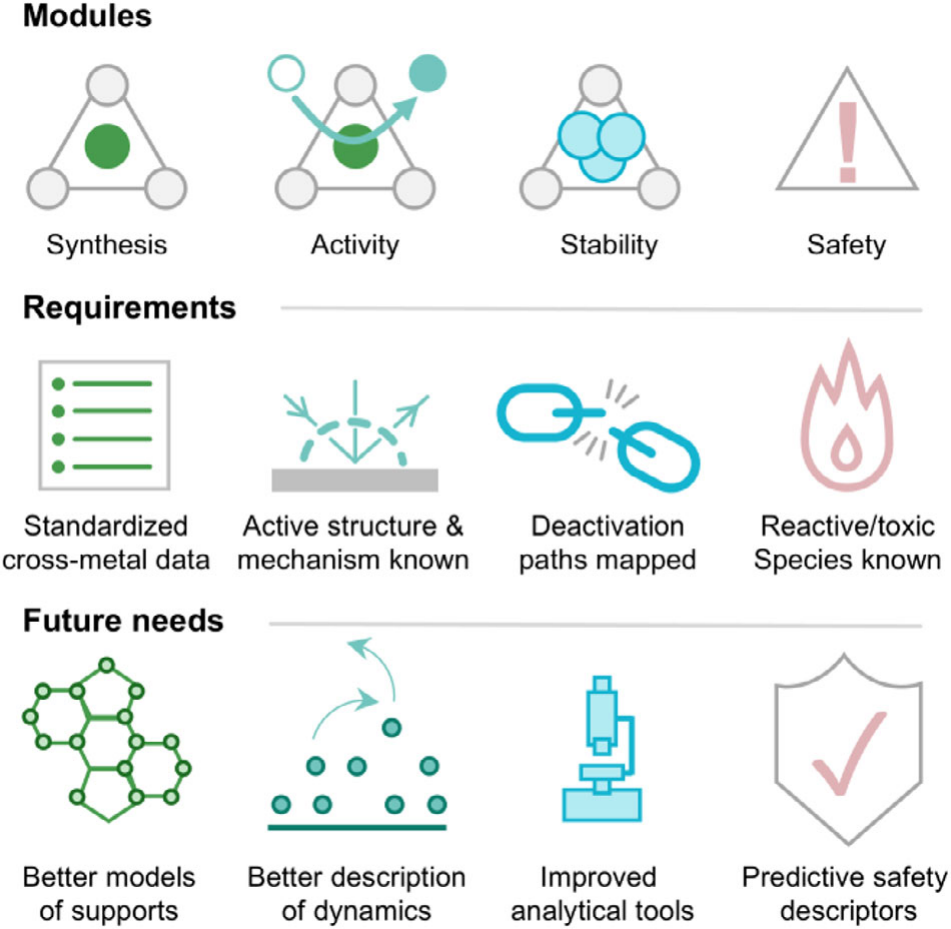
Directions for advancing predictive theory in single‐atom catalysis. Progress toward more predictive modeling requires improved representation of catalyst supports, enabled by machine‐learning interatomic potentials, molecular dynamics simulations, and operando approaches that capture site evolution under working conditions. Multimodal characterization is essential for resolving coupled deactivation pathways, while safety considerations must be addressed through descriptors of unstable or reactive intermediates. Comparative lifecycle benchmarks across these dimensions provide a basis for cross‐system evaluation and transferability.

Moving beyond thermodynamic descriptions toward explicitly dynamic or kinetic treatments will require substantially stronger experimental constraints and more faithful representations of catalyst supports, capable of capturing structural disorder, chemical heterogeneity, and site evolution under operating conditions. Progress in this direction hinges on tighter integration between theory and advanced, multimodal characterization. On the modeling side, this demands improved sampling strategies and representations of disordered materials that move beyond idealized representations. Machine‐learning interatomic potentials, molecular dynamics, and accelerated sampling techniques provide promising routes to extend the accessible configurational and chemical spaces (Figure 7). When combined with disciplined validation strategies, these developments can expand the scope of SAC modeling without sacrificing the transparency, control, and realism that are essential for theory to remain experimentally relevant.

## 5 Conclusion

A lifecycle‐aware framework based on thermodynamic ensembles provides a coherent way to interrogate single‐atom catalysts across synthesis, catalytic operation, deactivation, and safety. Within this framework, modeling decisions and approximations are made explicitly, starting from the thermodynamics of site formation during synthesis and propagated through subsequent stages. This allows the structural ensembles relevant to catalytic operation to be defined and evaluated against experimental observables, making explicit the assumptions and structural uncertainties that are often implicit in conventional single‐site representations.

For theory to be predictive in single‐atom catalysis, the thermodynamic description must explicitly account for ensembles of coordination environments and the dominant sources of variability, which in these systems are predominantly structural. Experimental benchmarking against independent observables then constrains these ensembles and bounds the associated uncertainty, defining the practical predictive scope of the model. Within such a calibrated framework, theory can guide catalyst development by identifying activity and stability regimes and contribute to the prioritization of catalyst compositions, coordination environments, and operating conditions for experimental investigation, with the achievable predictive resolution determined by the level at which uncertainty can be bounded.

More broadly, this perspective outlines a disciplined pathway for advancing theory in single‐atom catalysis across the field. As experimental datasets become more coherent and mechanistically resolved, the lifecycle‐oriented approach articulated here offers a transferable structure for deploying theory transparently and proportionately, allowing its predictive contribution to catalyst design to expand in step with experimental resolution.

## 6 Experimental Section/Methods

Density functional theory (DFT) simulations were performed on models of SAC using the Vienna ab initio Simulation Package (VASP 5.4.4) [47, 48]. The exchange–correlation energy was described with the Perdew–Burke–Ernzerhof (PBE) functional within the generalized gradient approximation (GGA) [49], and dispersion interactions were included through Grimme's DFT‐D3 scheme [50]. Core electrons were represented by the projector augmented wave (PAW) method [51, 52], and plane‐wave basis sets with a kinetic energy cutoff of 450 eV were used to describe valence states. Spin polarization was included where appropriate. Activated carbon supports were modeled as three‐layer 6 × 6 graphitic slabs separated by 12 Å of vacuum and sampled with a gamma‐centered 3 × 3 × 1 *k*‐point mesh. SAC were represented by placing the metal atom at the center of each cavity, and dipole corrections were applied to all slab models [53]. Structural relaxations were performed until the forces and energies converged below 10^−4^ and 10^−5^ eV for ionic and electronic steps, respectively. To evaluate precursor adsorption, solvent effects were included with the Solvation Model Based on Density (SMD), and entropic corrections were applied for gas‐phase species.

Physisorbed molecules were considered to preserve ∼2/3 of their gas phase entropy [54], as translation over the carbon plane was feasible (i.e., barrier lower than 0.7 eV). The stability of single atoms on the activated carbon support was assessed through formation energy calculations referenced to the most stable bulk metal phases, as reported in the Materials Project database.

## Funding

This study was created as part of NCCR Catalysis (grant number 225147), a National Centre of Competence in Research funded by the Swiss National Science Foundation.

## Conflicts of Interest

The authors declare no conflict of interest.

## Supporting information




**Supporting File**: advs75156‐sup‐0001‐SuppMat.pdf.

## Data Availability

The computational data generated in this study are available in the Supplementary Information and Source Data files, and have also been deposited in the Zenodo repository (https://doi.org/10.5281/zenodo.17232885) and the ioChem‐BD database (https://doi.org/10.19061/iochem‐bd‐1‐403) [55].
