# Supplementary material for: Toward Predictive Theory in Single‐Atom Catalysis
Source: Adv Sci (Weinh). 2026 Apr 3;13(27):e75156. doi: 10.1002/advs.75156 (PMC13170184; doi:10.1002/advs.75156)
Supplement: Supplementary file 1 — Supporting File: advs75156‐sup‐0001‐SuppMat.pdf. [file ADVS-13-e75156-s001.pdf]

Supporting Information

Toward Predictive Theory in Single-Atom Catalysis

Andrea Ruiz-Ferrando, Sharon Mitchell, Núria López, Javier Pérez-Ramírez**

Table of Contents

Supplementary Notes 1

Supplementary Tables 6

Supplementary Figures 19

Supplementary References 21

Note S1. Data Collection and Literature Analysis

We performed a systematic Scopus® literature search (accessed July 6, 2025) using “acetylene hydrochlorination” in titles, abstracts, or keywords, and “single atom” and “DFT” across all fields, to survey theoretical modeling practices for SAC. The initial query returned 115 publications, which were manually curated to exclude purely experimental studies without DFT modeling, homogeneous catalysts, metal-free catalysts, or metal nanoparticle catalysts, studies using non-carbon supports (e.g., oxides, zeolites, ceria), and review articles. After filtering, 46 studies were retained, all modeling studies of SAC, encompassing supported metal atoms with or without ligands.

Each publication was analyzed across four dimensions corresponding to lifecycle-related questions discussed in the main text: synthesis, activity, stability and safety, and validation. The analysis documents how modeling frameworks are constructed and implemented, without evaluating computational accuracy or the correctness of specific mechanistic conclusions.

Classification was performed through structured reading of the full manuscript, including supporting information. For each study, we identified where modeling decisions were described (Computational Methods, DFT sections, structural models, and supporting figures) and traced how these models were subsequently used in the Results and Discussion to interpret experiments, propose mechanisms, or analyze stability.

The assignment was based on three complementary sources of evidence within each paper: (i) descriptions of computational setup, (ii) the structures, pathways, or stability analyses explicitly evaluated in figures and tables, and (iii) the way in which theoretical results are connected to experimental observations. Each publication was assigned to one category per subdimension (e.g., site construction, model type, etc.). Classification criteria are summarized in **Table S3**, detailed results in **Table S4**, and aggregated outcomes in **Figure 1a**.

Note S2. Thermodynamic Modeling of Ensembles

To simulate the thermodynamics of synthesis and obtain ab initio representations of the active site, two key steps were considered: (i) adsorption of metal precursors on activated carbon and (ii) ligand removal upon thermal activation. These steps mirror the sequence of processes in dry and wet impregnation, where dissolved metal precursors are first deposited and adsorbed onto the carbon surface, and subsequently undergo thermal treatment that induces bond formation with the support while simultaneously driving ligand loss. Wet and dry impregnation are among the most widely employed synthesis routes due to their versatility and scalability.[1]

Activated carbon exhibits a variety of structural and point defects and can display a nearly continuous stoichiometry with diverse surface functionalities depending on the preparation atmosphere.[2,3] Characterization techniques provide only partial insight into such environments. For example, EXAFS cannot distinguish between C, N, and O ligands and yields averaged signals over different sites, limiting the identification of host cavities. To address this, we constructed structural models consistent with the stoichiometry and dominant cavity types inferred from characterization. O 1*s* XPS spectra were deconvoluted into C–O contributions (e.g., ether, alcohol) and C=O contributions (e.g., ketone, lactone, carbonyl), yielding an approximate 3:2 ratio of C–O to C=O groups.[2] In line with this analysis, representative functional groups (epoxide, hydroxyl, ketone, and carboxylic acid) were incorporated in diverse morphologies and compositions, both in the basal plane (B) and at the edge (E) of the carbon framework, generating a total of 8 coordination cavities (B-epo, B-2×epo, B-hyd, B-2×hyd, B-2×keto, B-4×keto, E-keto, E-carb, **Figure 3a**).[4,5]

The metal precursors employed were $HAuCl_4 \cdot xH_2O$ and $RuCl_3 \cdot xH_2O$, both dissolved in aqua regia, and $CuCl_2 \cdot 2H_2O$ and H_2PtCl_6, both dissolved in water.[5,6] To avoid ambiguities in reservoir definition and charge balancing, the simulated precursors were restricted to well-defined chlorinated species with consistent oxidation states: $AuCl_3$, $PtCl_4$ (reflecting the tendency of each metal to lose chlorine ligands in solution), $RuCl_3$ and $CuCl_2$. Thermally induced evolution was modeled as stepwise ligand removal from the adsorbed precursor species, with coordinated chlorides successively eliminated to generate a series of species with varying chlorination states. For each metal and each degree of chlorination, all reasonable adsorption configurations were systematically explored.

In the thermodynamic description of the synthesis stage, ensemble properties are evaluated as population-weighted averages based on equilibrium populations obtained from Boltzmann statistics using the formation energies of the structures (**Equation 1**),

$$P(\mathrm{MCl}_x^{\#}, i) = = \frac{e^{\frac{-\Delta G(\mathrm{MCl}_x^{\#}, i)}{k_B \cdot T}}}{\sum_{j=1}^{M} e^{\frac{-\Delta G(\mathrm{MCl}_x^{\#}, j)}{k_B \cdot T}}} \qquad \textbf{Equation 1}$$

where $P(\mathrm{MCl}_x^{\#})$ is the equilibrium probability of MCl_x species *i*, $\Delta G_{\mathrm{form}}(\mathrm{MCl}_x^{\#})$ is its Gibbs free formation energy, k_B the Boltzmann constant, *T* the temperature in K, and *M* the total number of species per metal.

To compensate for the structural bias of planar carbon models, which underestimate the energetic spread of adsorption environments, we evaluated Boltzmann populations by including all configurations within 0.5 eV of the minimum-energy state. This cutoff was chosen to reflect the typical uncertainty of DFT relative energies (approximately by a factor of two), ensuring that higher energy but still thermodynamically relevant configurations contribute to the ensemble. Under the assumption that the number of potential adsorption sites greatly exceeds the number of metal atoms introduced, consistent with 1 wt.% loading, this procedure yields relative populations of metal species accessible under synthesis conditions, thereby connecting computed thermodynamics to experimentally relevant speciation.

In contrast, catalytic activity and selectivity are not evaluated as population-weighted averages, since the turnover frequency of a given site does not necessarily scale with its thermodynamic population and highly active sites may contribute disproportionately even at low abundance. To account for this, catalytic descriptors are evaluated individually for each coordination environment. To keep the mechanistic analysis tractable while retaining thermodynamically meaningful structures, the synthesis-derived pool of coordination environments is reduced by retaining only sites with equilibrium populations larger than 2%. This threshold removes marginal configurations while preserving the dominant fraction of the ensemble, ensuring that the mechanistic analysis focuses on coordination environments that contribute meaningfully to the thermodynamic population.

Following the site evolution analysis described in the main text, this set of sites is updated to account for chlorination under reaction conditions. For each ex-situ coordination environment satisfying the minimum HCl dissociation criterion ($\Delta G_{\mathrm{diss}}(\mathrm{H}^{\#}\text{-}\mathrm{Cl}^{*}) < -0.4$ eV), the corresponding chlorinated configurations generated through stepwise chloride coordination

replace the original site. This energetic threshold ensures that chlorination is thermodynamically favored beyond typical DFT uncertainty, allowing only coordination environments with a clear driving force for chloride accumulation to contribute to the operando ensemble.

If multiple chlorination steps are accessible, sequentially chlorinated configurations are considered. No populations are recomputed at this stage, as the thermodynamic distribution derived from synthesis conditions is not expected to represent the relative contributions of sites under reaction conditions. Catalytic activity, stability, and safety analyses are evaluated on this updated ensemble of coordination environments.

For the analysis of activity, configurations with strongly endergonic dissociation ($\Delta G_{\mathrm{diss}}(H^{\#}\text{-}Cl^{*}) > 0.4$ eV) are excluded as turnover-relevant environments, as such energies indicate a clearly unfavorable thermodynamic driving force for HCl activation beyond typical DFT uncertainty, making their contribution to catalytic turnover unlikely under reaction conditions.

Note S3. Charge Polarization Ratio of Metal-Acetylide Bonds

The reactivity of the M–C bond in MCl_x–C≡C–H units depends strongly on how electrons are distributed between the metal and the acetylide. When electrons concentrate on the acetylide fragment, the bond becomes more polar and reactive, whereas a more balanced sharing of charge reflects covalent stabilization and reduced reactivity. To capture this distribution in a single measure, we defined the charge polarization ratio, $R_{pol}(MCl_x–C_2H^*)$, which condenses the redistribution of electron density during M–C bond formation in a single value for comparison across different metals (**Equation 2**):

$$R_{pol}(MCl_x - C_2H^*) = \frac{|\Delta Q_{CC}|}{|\Delta Q_{CC}| + |\Delta Q_M|} \quad \textbf{Equation 2}$$

where ΔQ_{CC} and ΔQ_M represent the net charge redistribution around the acetylide carbon and the metal atom, respectively. Values of $R_{pol}(MCl_x–C_2H^*)$ close to 1 indicate that the redistribution is concentrated at the acetylide, consistent with a more ionic and thus reactive bond. Lower values reflect a more balanced sharing of charge between carbon and metal, corresponding to covalent contributions.

ΔQ_{CC} and ΔQ_M are obtained by integrating the differential electron density within a fixed-radius sphere (**Equation 3**):

$$\Delta Q_x = \sum_{i \in V_x} \Delta\rho_i \Delta V \quad \textbf{Equation 3}$$

where X is the center of integration (metal or acetylide carbon), V_x the spherical region of integration centered at atom X, *i* the grid points inside that region, $\Delta\rho_i$ the differential charge density at grid point i, and ΔV the volume represented by a single grid point.

The differential charge density is obtained by subtracting the densities of the isolated fragments computed in the same supercell and grid to ensure consistency (**Equation 4**):

$$\Delta\rho = \rho_{MCl_x\text{-C≡C–H}} - \rho_{MCl_x} - \rho_{\text{C≡C–H}} \quad \textbf{Equation 4}$$

where $\rho_{MCl_x\text{–C≡C–H}}$ is the density of the full (chlorinated) metal–acetylide system, ρ_{MCl_x} that of the pristine (chlorinated) metal species, and $\rho_{\text{C≡C–H}}$ that of the isolated acetylide fragment.

Supplementary Tables

Table S1. Assessment of the suitability of different single-atom catalysis domains for quantitative theory–experiment comparison.[5-19] Colors show how well each domain meets the conditions required (green: comprehensive; yellow: partial; grey: absent).

Criteria	Electrocatalysis	Thermocatalysis			
	ORR, OER, HER, CO_2RR, NRR	CO_2 hydrogenation	Selective oxidation / halogenation	NH_3 synthesis / decomposition	Acetylene hydrochlorination
Industrial Relevance	Expanding field; stability and scalability remain bottlenecks	Emerging CCU route, some systems piloted	Established chemistry; proof-of-concept	Early stage; harsh conditions, complex kinetics	Industrially deployed SAC to replace $HgCl_2$
Publication volume[a]	7490	1967	774	544	73
Comparable cross-metal datasets	Non-standardized setups; no consensus testing protocols	No standardized datasets; supports/ conditions vary	Diverse systems; no unified kinetic datasets	Inconsistent metrics; lacking kinetic benchmarks	Quantitative multi-metal datasets with uniform conditions
In situ/ operando insights	Growing, but atomic scale insights lacking and realistic-potential studies remain scarce	Partial evidence. Persistent pressure gap; most insights from DFT or ex situ	Some insights from AP-XPS/DRIFTS/ ETEM; weak activity correlation	Few SAC spectra; DRIFTS/NEXAFS intermediates, no time-resolved data	Comprehensive operando XAS across metals/stages (synthesis/application)
Deactivation insights	Leaching/restructuring recognized, not yet monitored operando	Sintering/carbide formation known, not time-resolved	Over-oxidation and migration recognized, low quantitative linkage	Nitridation/hydride/ aggregation observed, kinetics unquantified	Distinct, spectroscopically validated modes
Mechanistic & theoretical tractability	Potential/solvent-dependent; standard DFT omits charge effects	Mechanism debated (CO_2 dissociation vs formate/RWGS); strong support effects	Multiple O-activation routes; open-shell systems challenge DFT	Associative/ dissociative routes and magnetism complicate modeling	Simple single-product network; DFT reproduces performance/spectra
Hazardous intermediates	Radical or peroxide species, gas evolution risks unstudied	Carbidic species potentially unstable, unstudied	Halogenated oxidants corrosive, toxic, unstudied	Nitrides or hydrides pyrophoric, unstudied	Metal acetylides explosive and studied

[a]Publication volume denotes the total count of peer-reviewed publications assigned to each category.

Table S2. Representative single-atom catalysts for the hydrogen evolution reaction (HER) and extent of spectroscopic verification and experimental comparability.

Catalytic system	Metal scope	Spectroscopic data fresh catalyst[a]	Spectroscopic data in-situ/operando[a]	Spectroscopic data used catalyst	Electrolyte[b]	Voltage range / V[c]	Stability test[d]	Ref.
PtSA/Ni-NiO-Ag	Single	XPS, XAS	No	No	alkaline/neutral	0.05 to −0.50	CV, CA	20
Pt/NiFe	Single	XPS, XAS	Yes (Raman)	Yes (XPS)	alkaline	0.05 to −0.50	CP	21
Pt/NC	Single	XPS, XAS	Yes (XAS)	Yes (XAS)	acidic	0.05 to −0.05	CV	22
Pt/Mo_2C-NC	Single	XPS, XAS	Yes (Raman)	Yes (XPS)	acidic/alkaline	0 to −0.20	CV, CP	23
Ru/N–$Ti_3C_2T_x$	Single	XPS, XAS	No	Yes (XPS)	multiple	0 to −0.20	CP	24
Co/PC	Single	Raman, XPS, XAS	No	No	acidic	0.05 to −0.80	CV, CP	25
Ir/C	Single	Raman, XPS, XAS, NMR	No	No	multiple	0 to −0.05	CA	26
TM/NC (V–Bi)	Multiple	Raman, XAS	No	No	acidic	0 to −0.20	CV	27
Pt/MoS_2	Single	XPS, XAS	No	No	acidic/alkaline	0.05 to −0.60	CV, CA	28
W/CoP	Single	XPS	No	No	multiple	0 to −0.60	CA	29
Gd/MoS_2	Single	Raman, XPS	No	Yes (XPS)	acidic/alkaline	0.1 to −0.6	CA	30
Pt/TiB_xO_Y	Single	Raman, XPS, XAS	No	No	acidic	0.2 to −0.4	CV, CA	31

[a]In-situ Raman measurements probe adsorbed intermediates rather than the catalyst directly. [b]Acidic: 0.5 M H_2SO_4; Alkaline: 1 M KOH; Neutral: 0.1-1 M PBS (phosphate-buffered saline); multiple: studies tested several media (acidic, alkaline, neutral, or seawater). [c]Referenced to the reversible hydrogen electrode (RHE). [d]Stability tests performed by cyclic voltammetry (CV), chronoamperometry (CA), or chronopotentiometry (CP).

Analysis of the most highly cited SAC studies on the hydrogen evolution reaction (>10 citations yr^{-1}) reveals limited comparability between datasets and few in-situ validations of active metal coordination environments. Most reports rely on ex-situ XPS/XAS analysis of fresh catalysts. While many studies employ comparable electrolytes, potential windows, and three-electrode configurations, differences in catalyst supports and durability testing protocols (varying in duration, current density, and potential control) hinder direct quantitative comparison.

Table S3. Classification scheme defining the conceptual categories used to analyze DFT modeling practices in studies of single-atom catalysts for acetylene hydrochlorination, including the criteria used to identify each category in the analyzed publications.

Dimension	Subdimension	Category	Definition	Identification in paper
Synthesis	Site construction	Sampling-based	Structural possibilities explored across motifs.	Multiple coordination motifs explicitly enumerated and tested.
		Precursor-based	Active-site model built from precursor species.	Use of precursor fragments directly as the catalytic site model.
		Spectroscopy-fitted	Active site constrained by experimental data.	Structure chosen to reproduce XAS coordination numbers.
	Model type	Periodic slab	Catalyst treated as extended periodic material.	Structure modeled as a periodic structure.
		Finite cluster	Catalyst represented as finite molecular fragment.	Cluster structure used as computational model.
	Site heterogeneity	Multi-site	Several candidate environments considered.	Calculations include more than one structural motif.
		Single-site	Only one active-site environment considered.	All calculations performed on a single structure.
	Site formation energy	Formation energies	Thermodynamic stability of sites compared.	Formation energies reported for alternative site motifs.
		Not evaluated	Relative stability between sites not analyzed.	No energetic comparison between candidate sites.
Activity	Site evolution	Structural evolution	Active-site structure allowed to evolve.	Possible restructuring of the site discussed in theoretical analysis.
		Ex-situ representation	Active-site structure assumed invariant.	Reaction mechanism evaluated on one predefined structure.
	Site contribution	Multi-site	Catalytic activity discussed on multiple sites.	Reaction pathways computed for several site motifs.
		Single-site	Catalytic activity attributed to one site.	Mechanism evaluated for a single site structure.
Stability and safety	Stability analysis	Deactivation modeling	Deactivation processes explicitly examined.	Simulations include sintering, poisoning, coking or others.
		Thermal stability	Stability discussed as energetic stability of the site.	Thermal stability evaluated via binding energies.
		Reaction-electronic	Stability inferred from reaction and electronics.	Discussed via reaction energies and electronic properties.
		Single-proxy inference	Stability inferred from reaction or electronics.	Discussed via reaction energies or electronic descriptors alone.
		Not assessed	Catalyst stability not addressed theoretically.	No stability-related discussion in the DFT section.
Validation	Validation reference	Hybrid	Multiple observable classes used for validation.	Both spectroscopic and catalytic metrics used for comparison.
		Performance-based	Validation based on activity or adsorption metrics.	Comparison made with activity, selectivity, or adsorption capacity.
		Spectroscopy-based	Validation based on spectroscopic observables.	Comparison made with XAS, XPS, or related measurements.
		Not performed	No theory-experiment comparison reported.	DFT results discussed independently from experiments.

Table S4. Classification of DFT studies on single-atom catalysts for acetylene hydrochlorination, where each publication (identified by DOI) is categorized according to the modeling assumptions used to construct, analyze, and validate the active-site models.

DOI	Category							
	Site construction	Model type	Site heterogeneity	Site formation energy	Site evolution	Site contribution	Stability analysis	Validation reference
10.1038/s41467-025-60169-7	Sampling	Periodic	Multi-site	Yes	Evolution	Single-site	Deactivation	Spectroscopy
10.1016/j.cjche.2024.11.007	Sampling	Cluster	Multi-site	Yes	Ex-situ	Single-site	Thermal	Performance
10.1016/j.apcata.2025.120113	Precursor	Cluster	Single-site	No	Ex-situ	Single-site	Not assessed	Performance
10.1039/D4CY01414F	Precursor	Cluster	Single-site	No	Ex-situ	Single-site	Single-proxy	Performance
10.1016/j.mcat.2024.114756	Spectroscopy	Cluster	Single-site	No	Ex-situ	Single-site	Not assessed	Performance
10.1002/anie.202501370	Sampling	Periodic	Multi-site	No	Ex-situ	Multi-site	Reaction-electronic	Performance
10.1002/aoc.7681	Precursor	Cluster	Single-site	No	Ex-situ	Single-site	Reaction-electronic	Hybrid
10.1021/acscatal.4c03533	Sampling	Periodic	Multi-site	No	Evolution	Single-site	Not assessed	Hybrid
10.1021/acs.iecr.4c00167	Precursor	Cluster	Single-site	No	Ex-situ	Single-site	Single-proxy	Hybrid
10.1039/D4ME00045E	Precursor	Cluster	Single-site	No	Ex-situ	Single-site	Single-proxy	Hybrid
10.1016/j.comptc.2023.114359	Sampling	Cluster	Multi-site	No	Ex-situ	Multi-site	Not assessed	Not performed
10.1016/j.apcata.2023.119382	Spectroscopy	Periodic	Single-site	Yes	Ex-situ	Single-site	Not assessed	Performance
10.1016/j.gee.2022.01.006	Spectroscopy	Cluster	Multi-site	No	Ex-situ	Multi-site	Single-proxy	Performance
10.1021/acscatal.3c01527	Spectroscopy	Periodic	Single-site	No	Ex-situ	Single-site	Single-proxy	Hybrid
10.1016/j.apcata.2023.119238	Precursor	Cluster	Single-site	No	Ex-situ	Single-site	Reaction-electronic	Performance
10.1007/s12274-023-5681-3	Precursor	Periodic	Single-site	No	Ex-situ	Single-site	Single-proxy	Hybrid
10.1002/adma.202211464	Sampling	Periodic	Multi-site	Yes	Evolution	Single-site	Thermal	Spectroscopy
10.1016/j.jcat.2023.04.005	Sampling	Cluster	Multi-site	Yes	Ex-situ	Single-site	Thermal	Hybrid
10.1016/S1872-2067(22)64204-9	Precursor	Cluster	Single-site	No	Ex-situ	Single-site	Single-proxy	Performance
10.1021/acssuschemeng.2c07478	Spectroscopy	Cluster	Single-site	No	Ex-situ	Single-site	Single-proxy	Performance

10.1021/acs.jpcc.2c06889	Sampling	Periodic	Multi-site	Yes	Evolution	Single-site	Thermal	Not performed
10.1038/s42004-021-00619-7	Sampling	Cluster	Multi-site	Yes	Ex-situ	Single-site	Thermal	Hybrid
10.1021/acs.iecr.2c03009	Precursor	Periodic	Single-site	Yes	Ex-situ	Single-site	Deactivation	Performance
10.1002/cctc.202200785	Precursor	Cluster	Multi-site	Yes	Ex-situ	Multi-site	Thermal	Performance
10.1039/D2CY00786J	Spectroscopy	Cluster	Single-site	No	Ex-situ	Single-site	Not assessed	Performance
10.1016/j.cjche.2021.04.026	Sampling	Cluster	Multi-site	No	Ex-situ	Multi-site	Not assessed	Performance
10.1039/D1QI01164B	Sampling	Periodic	Multi-site	Yes	Ex-situ	Single-site	Deactivation	Performance
10.1016/j.apcata.2021.118461	Precursor	Cluster	Single-site	No	Ex-situ	Single-site	Single-proxy	Performance
10.1039/D1CY01357B	Precursor	Cluster	Single-site	No	Ex-situ	Single-site	Not assessed	Performance
10.1021/acs.jpclett.1c01779	Spectroscopy	Periodic	Single-site	Yes	Ex-situ	Single-site	Single-proxy	Hybrid
10.1016/j.mcat.2021.111826	Sampling	Periodic	Multi-site	Yes	Ex-situ	Multi-site	Thermal	Not performed
10.1021/acsanm.1c00945	Sampling	Periodic	Multi-site	Yes	Ex-situ	Multi-site	Thermal	Not performed
10.1016/j.colsurfa.2021.126495	Sampling	Cluster	Multi-site	No	Ex-situ	Multi-site	Not assessed	Not performed
10.1016/j.colsurfa.2020.125230	Sampling	Cluster	Multi-site	No	Ex-situ	Multi-site	Not assessed	Not performed
10.1039/D0QI00694G	Precursor	Cluster	Single-site	No	Evolution	Single-site	Deactivation	Performance
10.1038/s41929-020-0431-3	Sampling	Periodic	Multi-site	Yes	Ex-situ	Multi-site	Deactivation	Hybrid
10.3390/catal9100808	Precursor	Cluster	Single-site	No	Ex-situ	Single-site	Not assessed	Not performed
10.1039/C9QI00904C	Sampling	Cluster	Single-site	No	Ex-situ	Single-site	Thermal	Not performed
10.1021/acs.jpcc.9b07557	Sampling	Periodic	Multi-site	Yes	Ex-situ	Multi-site	Thermal	Not performed
10.1016/j.apsusc.2019.02.007	Sampling	Periodic	Multi-site	No	Ex-situ	Multi-site	Not assessed	Not performed
10.1016/j.carbon.2019.01.102	Sampling	Periodic	Multi-site	No	Ex-situ	Multi-site	Not assessed	Hybrid
10.1016/S1872-2067(19)63271-7	Sampling	Cluster	Multi-site	Yes	Ex-situ	Single-site	Thermal	Hybrid
10.1039/C8SC03186J	Sampling	Periodic	Multi-site	Population	Ex-situ	Multi-site	Thermal	Hybrid
10.1016/j.jcat.2018.06.030	Spectroscopy	Periodic	Single-site	No	Ex-situ	Single-site	Thermal	Performance
10.1016/j.comptc.2018.03.015	Sampling	Cluster	Multi-site	Yes	Ex-situ	Multi-site	Single-proxy	Not performed
10.1016/j.catcom.2017.07.022	Spectroscopy	Periodic	Multi-site	No	Evolution	Single-site	Deactivation	Not performed

Table S5. Thermodynamic, statistical, and electronic descriptors for acetylene hydrochlorination, forming the fundamental basis of the framework.

Descriptor	Label	Interpretation	Computed as
Formation of metal sites[a]	$\Delta G_{form}(MCl_x^{\#})$[b,c]	Site formation	$G(MCl_x^{\#}) - G(C) - G(MCl_{ref}) + (ref - x)\cdot 0.5{*}G(Cl_2, g)$[d]
Probability of a metal site	$P(MCl_x^{\#})$	Site distribution	See **Note S2**
HCl dissociation	$\Delta G_{diss}(H^{\#}\text{-}Cl^*)$	HCl activation	$G(HCl^*) - G(MCl_x^{\#}) - G(HCl, g)$
C_2H_2 adsorption	$\Delta G_{ads}(C_2H_2^*)$	C_2H_2 interaction with metal	$G(C_2H_2^*) - G(MCl_x^{\#}) - G(C_2H_2, g)$
Competitive adsorption	$\Delta\Delta G_{comp}(H\#\text{-}Cl^*–C_2H_2^*)$	Reactant activation preference	$\Delta G_{diss}(HCl^*) - \Delta G_{ads}(C_2H_2^*)$
Stability of metal species	$\Delta G_{bind}(MCl_x^{\#})$	Volatilization/aggregation	$G(MCl_x^{\#}) - G(C) - G(MCl_x, g/bulk)$
Ionicity of acetylides	$R_{pol}(MCl_x–C_2H^*)$	Acetylide reactivity	See **Note S3**
C_2H_2 dissociation	$\Delta G_{diss}(C_2H^*\text{-}H^{\#})$	Acetylide formation	$G(C_2H^*\text{-}H^{\#}) - G(MCl_x^{\#}) - G(C_2H_2, g)$
C_2H_2 adsorption	$\Delta G_{ads}(C_2H_2^{\#})$	C_2H_2 activation and coking	$G(C_2H_2^*) - G(MCl_x^{\#}) - G(C_2H_2, g)$

[a]Metal sites include all the range of chlorination degrees considered for each metal. [b]* denotes adsorption on a metal site, and # adsorption on a carbon site. [c]x = 0–4. [d]ref = number of chlorine ligands in the metal precursor.

Table S6. Gibbs free adsorption energies ($\Delta G_{form}(MCl_x^{\#})$, in eV) of $AuCl_3$, $PtCl_4$, $RuCl_3$ and $CuCl_2$ species on the distinct cavities at 473 K, referenced to pristine cavities and to solvated molecular states, together with the corresponding Boltzmann populations ($P(MCl_x^{\#})$, as %) derived from these energies.

Cavity	Metal species							
	$AuCl_3$		$PtCl_4$		$RuCl_3$		$CuCl_2$	
	ΔG_{form}	P	ΔG_{form}	ΔG_{form}	ΔG_{form}	P	ΔG_{form}	P
B-epo	1.18	0.46	-[a]	-[a]	-[a]	-[a]	1.40	0.02
B-2×epo	0.69	40.54	0.22	23.29	−0.88	96.70	0.73	6.68
B-hyd	-[a]	-[a]	-[a]	-[a]	-[a]	-[a]	-[a]	-[a]
B-2×hyd	-[a]	-[a]	0.72	0.25	-[a]	-[a]	1.31	0.03
B-2×keto	0.90	5.89	1.28	0.00	0.79	0.00	0.99	0.65
B-4×keto	0.68	45.44	0.10	72.00	−0.51	3.20	0.46	82.20
E-keto	0.87	7.66	0.40	4.44	−0.14	0.11	0.69	10.01
E-carb	1.59	0.01	1.04	0.01	0.49	0.00	1.04	0.41

[a]Formation of volatile species.

Table S7. Gibbs free adsorption energies ($\Delta G_{form}(MCl_x^{\#})$, in eV) of $AuCl_x$, $PtCl_x$, $RuCl_x$ and $CuCl_x$ species (x = 0−4) on distinct cavities at 473 K in reference to pristine cavities, solvated metal precursors ($AuCl_3$, $PtCl_4$, $RuCl_3$ and $CuCl_2$) and Cl_2, together with the corresponding Boltzmann populations ($P(MCl_x^{\#})$, as %) derived from these energies.

Metal species	Cavity							
	B-2×epo		B-2×keto		B-4×keto		E-keto	
	ΔG_{form}	P	ΔG_{form}	P	ΔG_{form}	P	ΔG_{form}	P
$AuCl_3^{\#}$	0.69	0.40	0.90	0.06	0.68	0.44	0.87	0.07
$AuCl_2^{\#}$	0.24	25.49	1.04	0.02	0.14	63.30	0.34	9.86
$AuCl^{\#}$	0.97	0.03	1.09	0.01	0.71	0.34	1.05	0.02
$Au^{\#}$	2.84	0.00	2.30	0.00	0.94	0.04	2.24	0.00
$PtCl_4^{\#}$	0.22	1.97	1.28	0.00	0.10	6.10	0.40	0.38
$PtCl_3^{\#}$	0.46	0.22	1.10	0.00	0.28	1.11	0.59	0.07
$PtCl_2^{\#}$	0.05	9.56	0.82	0.01	−0.06	26.61	0.14	4.01
$PtCl^{\#}$	0.93	0.00	1.62	0.00	0.16	3.50	1.31	0.00
$Pt^{\#}$	3.14	0.00	3.26	0.00	−0.13	46.47	2.52	0.00
$RuCl_3^{\#}$	−0.88	85.89	0.79	0.00	−0.51	2.84	−0.14	0.09
$RuCl_2^{\#}$	−0.12	0.08	1.27	0.00	0.20	0.00	0.30	0.00
$RuCl^{\#}$	-[a]	-[a]	2.32	0.00	−0.66	11.09	1.69	0.00
$Ru^{\#}$	-[a]	-[a]	4.17	0.00	0.24	0.00	3.11	0.00
$CuCl_2^{\#}$	0.73	0.23	0.99	0.02	0.46	2.88	0.69	0.35
$CuCl^{\#}$	0.82	0.11	1.28	0.00	0.19	31.69	0.49	2.04
$Cu^{\#}$	2.51	0.00	2.11	0.00	0.12	62.70	1.82	0.00

[a]Formation of volatile species.

Table S8. Gibbs free energies for HCl dissociation (ΔG_{diss}($H^{\#}$-Cl*), in eV), C_2H_2 adsorption (ΔG_{ads}(C_2H_2*), in eV), and competitive adsorption between HCl and C_2H_2 (ΔΔG_{comp}($H^{\#}$-Cl*/C_2H_2*), in eV) on (chlorinated) metal species at 473 K.

Metal species	Cavity											
	B-2×epo			B-2×keto			B-4×keto			E-keto		
	HCl	C_2H_2	Comp[b]	HCl	C_2H_2	Comp	HCl	C_2H_2	Comp	HCl	C_2H_2	Comp
$AuCl_3^{\#}$	0.88	-[a]	-[c]	-[a]	-[a]	-[c]	0.55	-[a]	-[c]	0.68	-[a]	-[c]
$AuCl_2^{\#}$	0.01	-[a]	-[c]	−0.09	1.02	−1.11	−0.36	1.77	−2.14	0.01	0.67	−0.66
$AuCl^{\#}$	−0.36	0.39	−0.75	-[a]	-[a]	-[c]	−0.16	0.88	−1.04	−0.96	0.34	−1.29
$Au^{\#}$	-[a]	−1.34	-[c]	−0.72	−0.24	−0.48	−0.97	0.31	−1.28	−1.69	−0.87	−0.82
$PtCl_4^{\#}$	0.43	0.90	−0.46	2.34	−0.13	2.47	−0.20	0.99	−1.20	0.54	0.91	−0.37
$PtCl_3^{\#}$	0.29	0.06	−0.35	0.18	1.07	−0.89	−0.81	0.54	−1.35	−0.77	0.27	−1.03
$PtCl_2^{\#}$	0.48	1.14	−0.66	0.33	0.26	0.07	−0.18	1.09	−1.27	0.05	1.04	−0.99
$PtCl^{\#}$	−0.37	−0.63	0.26	−0.43	−0.15	−0.28	−0.07	0.09	−0.16	−1.56	−0.93	−0.62
$Pt^{\#}$	-[a]	−1.46	-[c]	−1.06	−0.82	−0.24	0.99	0.80	0.20	−1.32	−0.63	−0.69
$RuCl_3^{\#}$	0.21	0.92	−0.72	−0.04	0.49	−0.52	−0.74	0.67	−1.41	−0.81	0.33	−1.14
$RuCl_2^{\#}$	0.62	0.69	−0.06	0.02	0.43	−0.41	−1.16	0.56	−1.72	−0.88	0.48	−1.36
$RuCl^{\#}$	-[a]	-[a]	-[c]	0.39	−0.47	0.86	1.31	1.45	−0.14	−1.57	−0.94	−0.63
$Ru^{\#}$	-[a]	-[a]	-[c]	−0.80	−0.95	0.15	1.14	0.57	0.57	−1.27	−0.13	−1.14
$CuCl_2^{\#}$	0.56	1.25	−0.68	0.65	−[a]	-[c]	0.22	-[a]	-[c]	0.20	0.76	−0.55
$CuCl^{\#}$	0.01	0.75	−0.74	0.01	0.31	−0.30	0.40	1.17	−0.77	−0.23	0.70	−0.92
$Cu^{\#}$	-[a]	−0.91	-[c]	−0.28	−0.09	−0.19	−0.19	0.71	−0.90	−1.53	−0.42	−1.11

[a]No adsorption or formation of volatile species. [b]Competitive adsorption between hydrogen chloride and acetylene; negative values indicate preference for hydrogen chloride. [c]Not calculated due to missing at least one of the adsorbate configurations.

Table S9. Hydrogen chloride dissociation free energies (ΔG_{diss}($H^{\#}$-Cl*), in eV) for representative metal–chloride configurations compared with operando changes in metal-chlorine coordination (ΔCN) relative to the ex-situ state derived from EXAFS[6].

System	ΔCN (M-Cl) (operando-fresh)	Metal species	Cavity	ΔG_{diss}($H^{\#}$-Cl*)
Au_{SA}/AC	-0.5	$AuCl_2^{\#}$	B-2×epo	0.01
		$AuCl_2^{\#}$	B-4×keto	−0.34
		$AuCl_2^{\#}$	E-keto	0.01
Pt_{SA}/AC	0.4	$PtCl_4^{\#}$	B-4×keto	−0.20
		$PtCl_2^{\#}$	B-2×epo	0.48
		$PtCl_2^{\#}$	B-4×keto	−0.18
		$PtCl_2^{\#}$	E-keto	0.05
		$PtCl^{\#}$	B-4×keto	−0.07
		$Pt^{\#}$	B-4×keto	0.99
Ru_{SA}/AC	0.9	$RuCl_3^{\#}$	B-2×epo	0.21
		$RuCl_3^{\#}$	B-4×keto	−0.74
		$RuCl^{\#}$	B-4×keto	1.31
Cu_{SA}/AC	-0.2	$CuCl_2^{\#}$	B-4×keto	0.22
		$CuCl^{\#}$	B-4×keto	0.40
		$CuCl^{\#}$	E-keto	−0.23
		$Cu^{\#}$	B-4×keto	−0.19

[a]EXAFS conditions: ex-situ He (473 K); operando HCl/C_2H_2 mixture (473 K).

Table S10. Metal–support binding free energies of representative metal chloride species ($\Delta G_{bind}(MCl_x^{\#})$, in eV) referenced to bulk metal and gas-phase molecular states for Au, Pt, Ru, and Cu single-atom catalysts together with operando EXAFS metal–metal coordination numbers (CN(M–M))[6] used to assess aggregation tendencies under reaction conditions.

System	Operando CN (M-M)	Metal species	Cavity	$\Delta G_{bind}(MCl_x^{\#})$, bulk	$\Delta G_{bind}(MCl_x^{\#})$, gas
Au_{SA}/AC	2.7 ± 0.3	$AuCl_3^{\#}$	B-4×keto	0.04	−0.31
		$AuCl_2^{\#}$	B-2×epo	−0.24	−0.67
		$AuCl_2^{\#}$	E-keto	−0.13	−0.57
Pt_{SA}/AC	M-M not found	$PtCl_4^{\#}$	B-4×keto	-[a]	−0.51
		$PtCl_2^{\#}$	B-2×epo	−0.20	−2.04
		$PtCl_2^{\#}$	B-4×keto	−0.31	−2.15
		$PtCl_2^{\#}$	E-keto	−0.10	−1.94
		$PtCl^{\#}$	B-4×keto	-[a]	-[a]
		$Pt^{\#}$	B-4×keto	−0.39	−6.15
Ru_{SA}/AC	0.2 ± 0.3	$RuCl_4^{\#}$	B-4×keto	-[a]	-[a]
		$RuCl_3^{\#}$	B-2×epo	−0.68	−2.36
		$RuCl^{\#}$	B-4×keto	-[a]	-[a]
Cu_{SA}/AC	M-M not found	$CuCl_2^{\#}$	B-4×keto	−0.29	−0.65
		$CuCl^{\#}$	B-4×keto	−0.76	−2.12
		$CuCl^{\#}$	E-keto	−0.46	−1.82
		$Cu^{\#}$	B-4×keto	−1.10	−4.33

[a]Bulk phase lacks an experimental reference, or the molecular reference would be charged.
[b]Formation of volatile species.

Table S11. Acetylene adsorption free energies on carbon species adjacent to representative metal chloride sites ($\Delta G_{ads}(C_2H_2^{\#})$, in eV) for Au, Pt, Ru, and Cu SAC, along with the specific surface area of fresh and spent catalysts measured by BET[5].

System	ΔS_{BET} / $m^2\ g^{-1}$	Metal species	Cavity	$\Delta G_{ads}(C_2H_2^{\#})^a$
Au_{SA}/AC	−193	$AuCl_3^{\#}$	B-4×keto	−0.92
		$AuCl_2^{\#}$	B-2×epo	-[a]
		$AuCl_2^{\#}$	E-keto	−0.41
Pt_{SA}/AC	−153	$PtCl_4^{\#}$	B-4×keto	−0.52
		$PtCl_2^{\#}$	B-2×epo	-[a]
		$PtCl_2^{\#}$	B-4×keto	−0.36
		$PtCl_2^{\#}$	E-keto	−0.33
		$PtCl^{\#}$	B-4×keto	0.22
		$Pt^{\#}$	B-4×keto	0.81
Ru_{SA}/AC	−788	$RuCl_4^{\#}$	B-4×keto	−0.61
		$RuCl_3^{\#}$	B-2×epo	-[b]
		$RuCl^{\#}$	B-4×keto	1.25
Cu_{SA}/AC	−1	$CuCl_2^{\#}$	B-4×keto	−0.53
		$CuCl^{\#}$	B-4×keto	−0.67
		$CuCl^{\#}$	E-keto	−0.59
		$Cu^{\#}$	B-4×keto	−0.15

[b]Formation of volatile species.

Table S12. Acetylene dissociation free energies, $\Delta G_{diss}(C_2H^*\text{-}H^\#)$, in eV, and charge polarization ratios, $R_{pol}(MCl_x–C_2H^*)$ for representative metal–chloride coordination environments of Au, Pt, Ru, and Cu SACs together with reference charge polarization ratios for their reference metal acetylide compounds (R_{pol} (M_2C_2)) used to contextualize the ionic character of the metal–acetylide interaction and its potential safety implications.

System	$R_{pol}(M_2C_2)$	Metal species	Cavity	$\Delta G_{diss}(C_2H^*\text{-}H^\#)$	$R_{pol}(MCl_x–C_2H^*)$
Au_{SA}/AC	0.44	$AuCl_3^\#$	B-4×keto	0.81	0.88
		$AuCl_2^\#$	B-2×epo	0.36	0.66
		$AuCl_2^\#$	E-keto	0.36	0.60
Pt_{SA}/AC	0.25	$PtCl_4^\#$	B-4×keto	−0.46	0.70
		$PtCl_2^\#$	B-2×epo	0.67	0.66
		$PtCl_2^\#$	B-4×keto	0.14	0.65
		$PtCl_2^\#$	E-keto	0.82	0.64
		$PtCl^\#$	B-4×keto	−0.65	0.70
		$Pt^\#$	B-4×keto	1.27	0.76
Ru_{SA}/AC	0.50	$RuCl_4^\#$	B-4×keto	0.07	0.96
		$RuCl_3^\#$	B-2×epo	-[b]	-[b]
		$RuCl^\#$	B-4×keto	1.13	0.57
Cu_{SA}/AC	0.56	$CuCl_2^\#$	B-4×keto	0.56	0.72
		$CuCl^\#$	B-4×keto	0.46	0.88
		$CuCl^\#$	E-keto	0.49	0.63
		$Cu^\#$	B-4×keto	0.83	0.94

Supplementary Figures

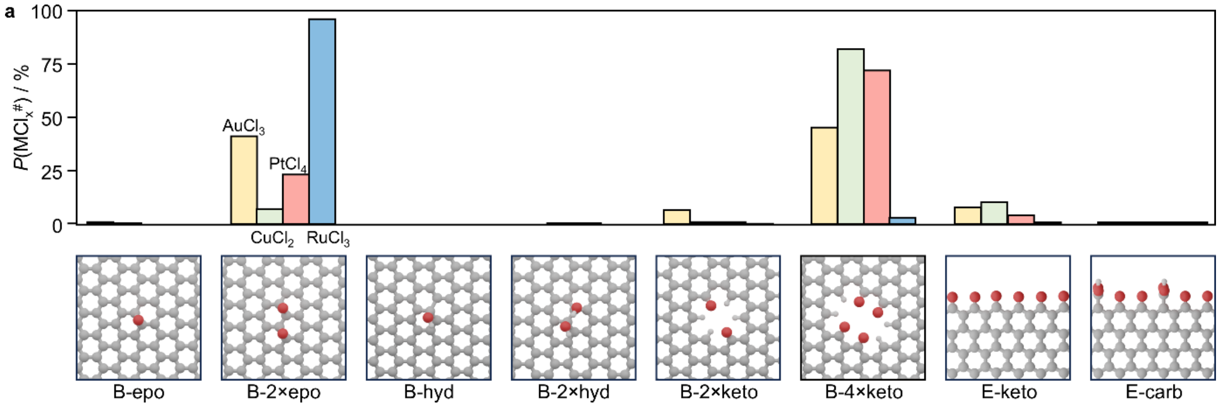

Figure S1. Boltzmann populations of metal–chloride species (MCl_x*) obtained from adsorption of $AuCl_3$, $PtCl_4$, $RuCl_3$, and $CuCl_2$ precursors on representative activated carbon motifs, illustrating how synthesis conditions translate into distributions of accessible sites. Structures below depict representative anchoring environments.

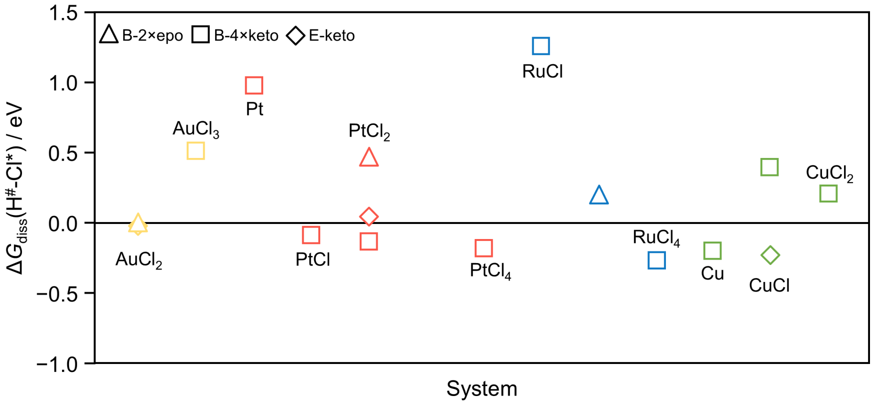

Figure S2. Hydrogen chloride dissociation free energies (ΔG_{diss}(H$^{\#}$-Cl*), in eV) for representative metal–chloride species sampled in the ensemble under reaction conditions.

Supplementary References

1 Kaiser, S. K., Chen, Z., Faust Akl, D., Mitchell, S. & Pérez-Ramírez, J. Single-atom catalysts across the periodic table. *Chem. Rev.* **120**, 11703–11809 (2020).

2 Kaiser, S. K. et al. Design of carbon supports for metal-catalyzed acetylene hydrochlorination. *Nat. Commun.* **12**, 4016 (2021).

3 Koppe, J. et al. Coordination environments of Pt single-atom catalysts from NMR signatures. *Nature* **642**, 613–619 (2025).

4 Faust Akl, D. et al. Reaction-induced formation of stable mononuclear Cu(I)Cl species on carbon for low-footprint vinyl chloride production. *Adv. Mater.* **35**, e2211464 (2023).

5 Kaiser, S. K. et al. Performance descriptors of nanostructured metal catalysts for acetylene hydrochlorination. *Nat. Nanotechnol.* **17**, 606–612 (2022).

6 Giulimondi, V. et al. Evidence of bifunctionality of carbons and metal atoms in catalyzed acetylene hydrochlorination. *Nat. Commun.* **14**, 5557 (2023).

7 Chen, Z., Wang, S., Zhao, J., Lin, R. Advances in single-atom-catalyzed acetylene hydrochlorination. *ACS Catal.* **14**, 965–980 (2024).

8 Yao, C. et al. Recent advances in carbon dioxide selective hydrogenation and biomass valorization via single-atom catalysts. *Res. Chem. Mater.* **2**, 189–207 (2023).

9 Choudhary, N., Nabeela, K., Mate, N. Recent advances in CO_2 hydrogenation to methane using single-atom catalysts. *RSC Sustain.* **2**, 1179–1201 (2024).

10 Peng, X et al. Single-atom and cluster catalysts for thermocatalytic ammonia synthesis at mild conditions. *Chem. Sci.* **15**, 5897–5915 (2024).

11 Yang, Y. et al. Recent advances in single-atom catalysts for electrochemical nitrate reduction to ammonia. *J. Environ. Chem. Eng.* **13**, 115144 (2025).

12 Saini, P. et al. Advances in ammonia decomposition catalysis: a comprehensive analysis of nanoparticle, single-atom, and metal cluster catalysts. *Chem. Commun.* **61**, 6027–6054 (2025).

13 Zhang, J. et al. Advances in CP catalytic oxidation on typical noble metal catalysts: Mechanism, performance and optimization. *Chem. Eng. J.* **495**, 153523 (2024).

14 Wei, K., Wang, X., Ge, J. Towards bridging thermo/electrocatalytic CO oxidation: from nanoparticles to single atoms. *Chem. Soc. Rev.,* **53**, 8903–8948 (2024).

15 Yu, X. et al. Single-atom catalysts: Preparation and applications in environmental catalysis. *Catalysts* **22**, 1239 (2023).

16 Li J. et al. Challenges and perspectives of single-atom-based catalysts for electrochemical reactions. *JACS Au* **3**, 736–755 (2023).

17 Dobrota, A. S., Pašti, I. A. Single-atom catalysts: Are you really single? *J. Phys. Chem. Lett.* **16**, 77−86 (2025).

18 Wang, Y. et al. Advanced electrocatalysts with single-metal-atom active sites. *Chem. Rev.* **120**, 12217−12314 (2020).

19 Jia, C. et al. Challenges and opportunities for single-atom electrocatalysts: From lab-scale research to potential industry-level applications. *Adv. Mater.* **36**, 2404659 (2024).

20 Zhou, K. L. et al. Platinum single-atom catalyst coupled with transition metal/metal oxide heterostructure for accelerating alkaline hydrogen evolution reaction. *Nat. Commun.* **12**, 3783 (2021).

21 Zhang, T. et al. Pinpointing the axial ligand effect on platinum single-atom-catalyst towards efficient alkaline hydrogen evolution reaction. *Nat. Commun.* **13**, 6875 (2022).

22 Li, J. et al. Unveiling the nature of Pt single-atom catalyst during electrocatalytic hydrogen evolution and oxygen reduction reactions. *Small* **17**, 2007245 (2021).

23 Yue, C. et al. Hierarchically stabilized Pt single-atom catalysts induced by an atomic substitution strategy for an efficient hydrogen evolution reaction. *Energy Environ. Sci.* **17**, 5227–5240 (2024).

24 Liu, H. et al. Single-atom Ru anchored in nitrogen-doped MXene ($Ti_3C_2T_x$) as an efficient catalyst for the hydrogen evolution reaction at all pH values. *J. Mater. Chem. A.* **8**, 24710–24717 (2020).

25 Huang, Y. et al. Phosphorus-modified cobalt single-atom catalysts loaded on crosslinked carbon nanosheets for efficient alkaline hydrogen evolution reaction. *Nanoscale* **15**, 3550–3559 (2020).

26 Liu, C. et al. Ir single atom catalyst loaded on amorphous carbon materials with high HER activity. *Adv. Sci.* **9**, 2105392 (2022).

27 Lai, W. H. et al. General synthesis of single-atom catalysts for hydrogen evolution reactions and room-temperature Na-S batteries. *Angew. Chem. Int. Ed.* **59**, 22171–22178 (2020).

28 Zhu, J. et al. Defect-assisted anchoring of Pt single atoms on MoS_2 nanosheets produces high-performance catalyst for industrial hydrogen evolution reaction. *Small* **18**, 2104824 (2022).

29 Wu, J. et al. Single-atom tungsten-doped CoP nanoarrays as a high-efficiency pH-universal catalyst for hydrogen evolution reaction. *ACS Sustain. Chem. Eng.* **8**, 14825–14832 (2020)

30 Zeng, W. et al. Atomic magnetic heating effect enhanced hydrogen evolution reaction of Gd@MoS_2 single-atom catalysts. *Small* **19**, 2206155 (2023).

31 Cheng, X. et al. Ligand charge donation-acquisition balance: A unique strategy to boost single Pt atom catalyst mass activity toward the hydrogen evolution reaction. *ACS Catal.* **12**, 5970–5978 (2022).
